# Clinical utility of the gastrointestinal dysfunction score versus acute gastrointestinal injury grade for short-term risk stratification in critically ill patients: evidence from two prospective cohorts

**DOI:** 10.3389/fnut.2026.1870956

**Published:** 2026-06-10

**Authors:** Yanhua Li, Youquan Wang, Yuhan Zhang, Feng Zhang, Lu Ke, Dong Zhang

**Affiliations:** 1 Department of Critical Care Medicine, The First Hospital of Jilin University, Changchun, China; 2 Department of Critical Care Medicine, Jinling Hospital, Medical School of Nanjing University, Nanjing, Jiangsu, China; 3 National Institute of Healthcare Data Science, Nanjing University, Nanjing, Jiangsu, China

**Keywords:** acute gastrointestinal injury, clinical outcomes, critically ill patients, gastrointestinal, gastrointestinal dysfunction score

## Abstract

**Objectives:**

To compare whether GIDS or AGI grade is more strongly associated with short-term outcomes in critically ill patients.

**Methods:**

We conducted a secondary analysis of two prospectively collected cohorts. The primary analyses was conducted using the single-center FJLU cohort of critically ill adults with organ failure (SOFA ≥2 within 24 h of ICU admission), and externally validated using the multicentre NEED trial cohort. The primary outcomes were 28-day mortality and ICU-free days to day 28. Associations with mortality were assessed using Cox models, discrimination by ROC curves with the DeLong test, and trends in ICU-free days using the Jonckheere–Terpstra test.

**Results:**

A total of 1,995 patients were included (FJLU *n* = 1,091; NEED *n* = 904), with 28-day mortality of 20.2 and 13.4%, respectively. Both AGI grade and GIDS were independently associated with higher 28-day mortality [FJLU: HR 1.45 (1.24–1.70) for AGI; 1.49 (1.27–1.75) for GIDS, per point increase]. Overall discrimination was similar, but GIDS performed better in more severely ill patients (APACHE II ≥ 15 or SOFA ≥8). GIDS also showed a more consistent inverse association with ICU-free days. Findings were validated in the NEED cohort.

**Conclusion:**

Both GIDS and AGI were associated with short-term outcomes in critically ill patients, with comparable overall prognostic performance. GIDS may provide additional value in patients with greater illness severity.

**Clinical trial registration:**

Identifier ChiCTR2300071370; ISRCTN12233792.

## Graphical abstract

**Figure:**
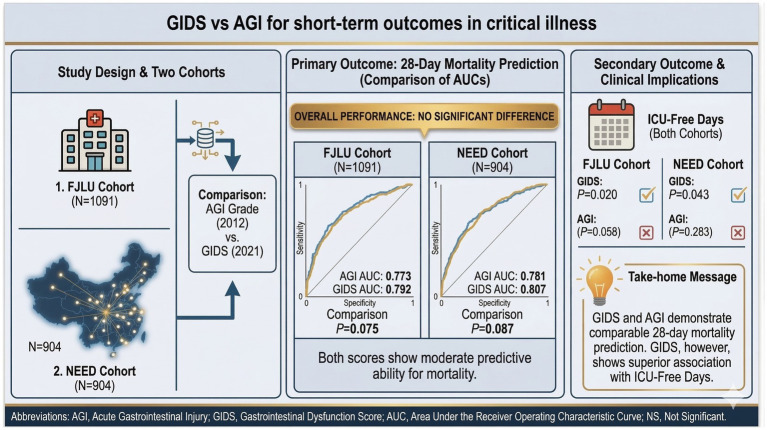

## 1 Background

The gastrointestinal tract is significantly affected during critical illness (1–4). Gastrointestinal dysfunction occurs in up to 60% of patients in the intensive care unit (ICU) and may adversely affect both short-term outcomes and the delivery and tolerance of nutritional therapy (5, 6). Despite growing recognition of the gut as a contributor to multiple organ dysfunction (1, 2, 7–11), gastrointestinal dysfunction is not included in commonly used ICU organ failure scoring systems, including updated versions of the Sequential Organ Failure Assessment (SOFA) score (12). One major reason is the lack of a reproducible, standardized, and bedside-feasible assessment tool (13). Early identification of clinically relevant gastrointestinal dysfunction may therefore be useful not only for risk stratification, but also for identifying patients who may require closer monitoring and more individualized nutritional support. A practical and clinically actionable gastrointestinal scoring system is therefore needed to improve bedside assessment and to inform supportive care planning in critically ill patients (5, 14, 15).

In 2012, the European Society of Intensive Care Medicine (ESICM) introduced the concept of acute gastrointestinal injury (AGI) (16) to assess and categorize gastrointestinal dysfunction in critically ill patients. However, the originally proposed AGI was largely descriptive and was not widely used in either clinical practice or research settings. The major limitation of AGI is its reliance on subjective clinical assessment and on feeding intolerance, a poorly standardized concept that varies according to local feeding practices (17). To address these shortcomings, Annika Reintam Blaser and colleagues developed the Gastrointestinal Dysfunction Score (GIDS) in 2021 (11), aiming to enable bedside assessment using more objective measurements and to provide a robust tool for evaluating gastrointestinal function in critically ill patients (18). At present, AGI and GIDS are the two most widely accepted approaches for gastrointestinal dysfunction scoring in critical care; however, whether GIDS offers clear superiority over AGI remains uncertain.

Existing evidence is inconsistent. Liu et al. (1) reported that GIDS had predictive value for 28-day mortality and achieved discrimination approximating that of Acute Physiology and Chronic Health Evaluation II (APACHE II) and SOFA, whereas AGI performed less well. By contrast, Shen et al. (19) found that GIDS was an unreliable predictor of 28-day mortality and that prognostic accuracy was comparable between AGI and GIDS. Both studies were limited by relatively small sample sizes; therefore, a large-scale, adequately powered evaluation is warranted to clarify differences between the two systems (20). In this study, we systematically compared the associations of AGI and GIDS with clinically important outcomes in two large, independent cohorts of critically ill patients. Our findings may help guide the selection of gastrointestinal assessment tools in the ICU and inform clinical decision-making in critically ill patients.

## 2 Methods

### 2.1 Data sources

The primary analysis used data from a single-center, prospective observational cohort conducted in the ICU of the First Hospital of Jilin University (FJLU) between May 2023 and April 2024 [FJLU Cohort Dataset (21)]. The study was approved by the Ethics Committee of the First Hospital of Jilin University and registered at the Chinese Clinical Trial Registry (ChiCTR2300071370) https://www.chictr.org.cn/hvshowproject.html?id=234518&v=1.2. To enhance the robustness and generalizability of our findings, we performed external validation using individual participant data from a multicenter, cluster-randomized controlled trial conducted in 2022 across 97 ICUs in China [NEED Trial Dataset (22), ISRCTN12233792], https://www.isrctn.com/ISRCTN12233792?q=ISRCTN12233792&filters=&sort=&offset=1&totalResults=1&page=1&pageSize=10 accessed under a formal data use agreement with the principal investigator.

Inclusion criteria were: (1) age ≥18 years; (2) diagnosis of AGI according to the 2012 ESICM (16); and (3) the presence of one or more organ failures within 24 h of ICU admission (SOFA≥2 for any single organ system). Exclusion criteria were: (1) receiving palliative care; (2) expected death within 72 h; (3) oral feeding; (4) pregnancy; (5) missing clinical data, including insufficient clinical data to evaluate the GIDS; (6) loss to follow-up.

### 2.2 Data collection and related scoring

Clinical data collected for enrolled patients included baseline characteristics (age, height, weight, primary diagnosis) and gastrointestinal symptoms (abdominal distension, vomiting, bowel sounds, stool frequency and volume, and intra-abdominal pressure [IAP]). Within the first 24 h after ICU admission, APACHE II, SOFA, and modified Nutrition Risk in the Critically Ill (mNUTRIC) scores were calculated using the worst recorded values. Additional variables included mechanical ventilation, the highest lactate level within 24 h, 28-day mortality, and ICU-free days to day 28.

AGI grade was determined by the attending ICU team within 24 h according to the 2012 ESICM recommendations (Supplementary Table S1) and subsequently reviewed by a dedicated researcher (Y. Zhang); discrepancies were resolved by consensus. GIDS was assigned according to the criteria proposed by Reintam Blaser et al. (11) (Supplementary Table S2) and independently evaluated by a trained researcher (Y. Li).

The NEED Trial dataset was provided by the Chinese Critical Care Nutrition Therapy Group (CCCNTG). For this dataset, GIDS were retrospectively reconstructed by Y. Li from prospectively collected clinical data because prospective bedside GIDS assessment was not part of the original trial protocol. GIDS reconstruction was based on available information on gastrointestinal symptoms (vomiting/regurgitation, bowel sounds, stool frequency and volume, gastrointestinal bleeding, abdominal distension), gastric residual volume (GRV), IAP, interruptions of nutrition, and use of prokinetic agents (Supplementary Table S3). When specific variables (e.g., GRV, IAP) were unavailable, GIDS was calculated from the remaining components. All final assessments were entered into an electronic database and locked before analysis.

### 2.3 Endpoints

The primary endpoint was 28-day mortality after ICU admission. The secondary endpoint was ICU-free days to day 28(the number of days alive and free from the ICU within the first 28 days).

### 2.4 Sample size consideration

Sample size was calculated in PASS 2023 (version 23.0.2) using the procedure for comparing two ROC curves. Based on previous studies, we assumed AUCs of 0.672 for AGI and 0.743 for GIDS in predicting 28-day mortality (1, 19).^.^ Using a two-sided Z-test with a Type I error rate of 0.05 and 90% power, a total sample size of 680 patients was required.

### 2.5 Statistical analysis

Continuous variables were expressed as mean ± standard deviation (SD) for normally distributed data and as median (interquartile range, IQR) for non-normally distributed data, as assessed by the Shapiro–Wilk test. Between-group differences were evaluated using the Student’s t test for normally distributed variables or the Mann–Whitney U test for non-normally distributed variables. Categorical variables were summarized as counts (percentages) and compared using the *χ*^2^ test or Fisher’s exact test, as appropriate. For ordered categories, trends were assessed using the Cochran–Armitage test for trend in proportions.

Patients with missing variables required for AGI grade or GIDS assessment were excluded from the corresponding analyses. In the NEED cohort, several key gastrointestinal variables required for GIDS reconstruction were unavailable rather than partially missing. Therefore, multiple imputation was not considered appropriate, and a complete-case analysis approach was adopted for the validation analyses.

We used Cox proportional hazards models to examine the association of AGI and GIDS with 28-day mortality. Hazard ratios (HRs) were estimated per 1-point and 1-standard deviation (SD) increase in each score. Variables with *p* < 0.10 in univariable analyses were entered into multivariable models. Multicollinearity was assessed using variance inflation factors (VIFs); variables with VIF ≥ 5 (tolerance < 0.2) were excluded. Final models yielded adjusted HRs with 95% confidence intervals (CIs). To compare the per-point effects of AGI and GIDS, we fitted a combined model and used a Wald test based on the covariance matrix of the coefficients. The proportional hazards assumption was checked using Schoenfeld residuals.

The discriminative performance of AGI grade and GIDS for 28-day mortality was assessed using receiver operating characteristic (ROC) curves and the area under the curve (AUC). AUCs with 95% CIs were estimated using the pROC package and compared between AGI grade and GIDS using DeLong’s test (23). Calibration for 28-day mortality was evaluated with calibration plots and the Hosmer–Lemeshow goodness-of-fit test. To assess the discriminative performance of AGI grade and GIDS for 28-day mortality across clinically relevant subgroups, we performed subgroup analyses in both the FJLU and NEED cohorts. ICU-free days to day 28 were analysed using the Jonckheere–Terpstra test. Multivariable linear regression with Wald tests was additionally performed as an adjusted analysis to examine whether the associations remained after controlling for potential confounders. Model assumptions were checked using residual diagnostics, and heteroscedasticity-robust standard errors (HC3) were applied when appropriate.

All analyses were performed using R software (version 4.4.2; R Foundation for Statistical Computing, Vienna, Austria) within RStudio (version 2024.12.1 + 563; Posit Software, Boston, MA, USA). Two-sided *p* values < 0.05 were considered statistically significant.

## 3 Result

### 3.1 Clinical and demographic data

**Figure 1:**
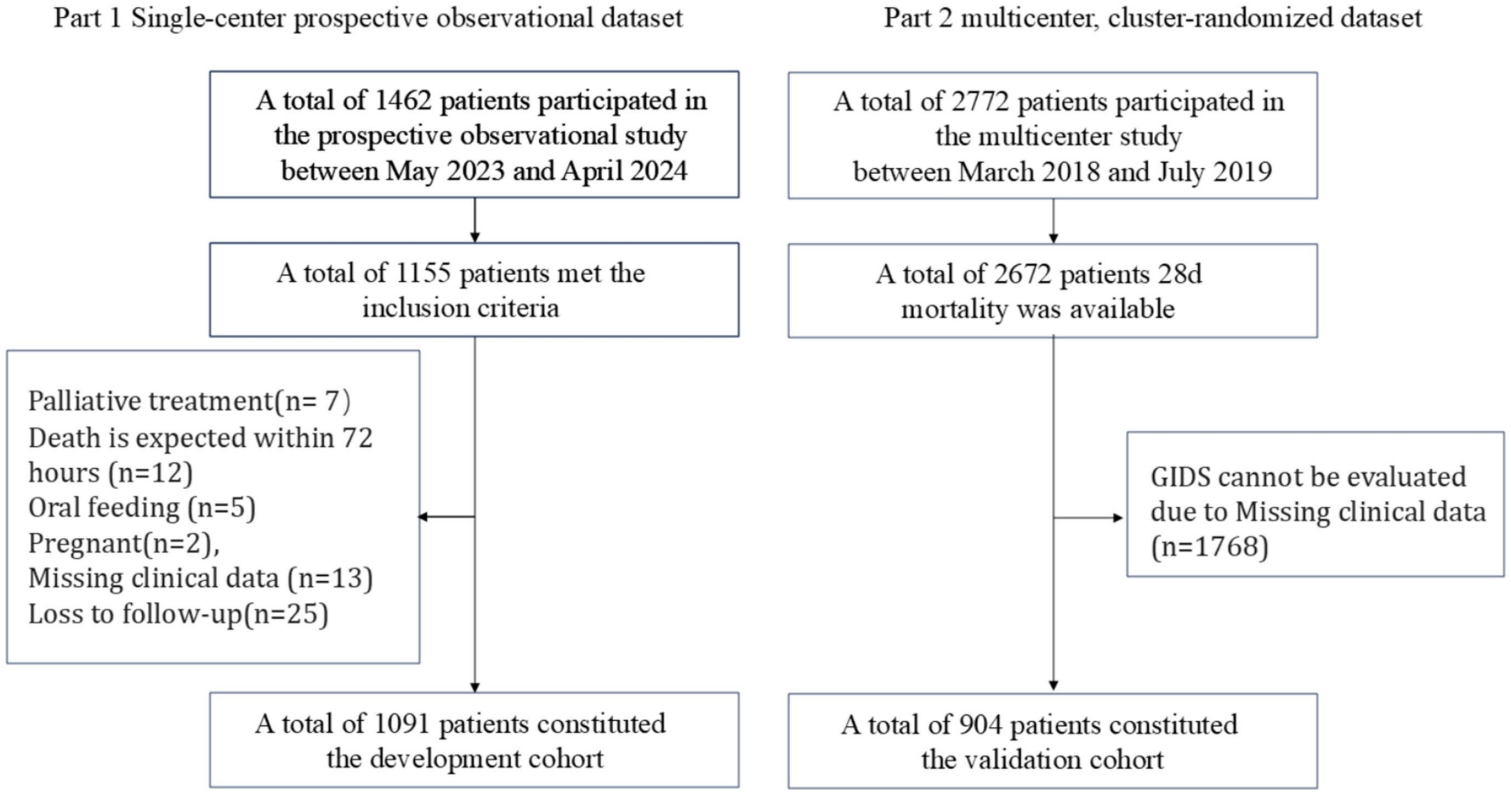
Flowchart of inclusion and exclusion for two datasets. GIDS, Gastrointestinal Dysfunction Score.

During the study period, 1,462 patients were screened, and 1,155 met the inclusion criteria. Of these, 39 were then excluded. In addition, 25 patients were lost to follow-up. The final analysis, therefore, included 1,091 patients. Of the 2,772 patients enrolled in the NEED trial, 1,768 were excluded because of missing variables required for GIDS reconstruction, leaving 904 patients in the final validation cohort. The flowchart of study patients is shown in Figure 1. The 28-day mortality rates were 20.2 and 13.4%, respectively. The clinical and demographic data for the two datasets are shown in Table 1.

**Table 1:** Baseline clinical and demographic characteristics of the two cohorts.

Variables	FJLU cohort dataset (*n* = 1,091)				NEED Trial Dataset (*n* = 904)			
	Total (*n* = 1,091)	Death (*n* = 221)	Survival (*n* = 870)	*p*-value	Total (*n* = 904)	Death (*n* = 121)	Survival (*n* = 783)	*p*-value
Age, mean (SD), y	62.5 (15.3)	64.3 (15.4)	62.0 (15.3)	0.049	63.2 (17.4)	66.6 (16.2)	62.7 (17.5)	0.021
Gender, No. (%)				0.913				0.620
Male	665 (61.0%)	134 (60.6%)	531 (61.0%)		608 (67.3%)	79 (65.3%)	529 (67.6%)	
Female	426 (39.0%)	87 (39.4%)	339 (39.0%)		296 (32.7%)	42 (34.7%)	254 (32.4%)	
BMI, mean (SD)	23.2 (3.9)	22.8 (4.0)	23.2 (3.9)	0.110	22.5 (3.1)	21.4 (2.9)	22.7 (3.1)	<0.001
Primary diagnosis, No. (%)								
Neurologic	220 (20.2%)	45 (20.4%)	175 (20.1%)		279 (30.9%)	31 (25.6%)	248 (31.7)	
Cardiovascular	66 (6.0%)	10 (4.5%)	56 (6.4%)		200 (22.1%)	32 (26.4%)	168 (21.5%)	
Respiratory	470 (43.1%)	101 (45.7%)	369 (42.4%)		292 (32.3%)	52 (43.0%)	240 (30.7%)	
Multi trauma	73 (6.7%)	12 (5.4%)	61 (7.0%)		51 (5.6%)	1 (0.8%)	50 (6.4%)	
Others	262 (24.0%)	53 (24.0%)	209 (24.0%)		82 (9.1%)	5 (4.1%)	77 (9.8%)	
APACHE II, median (IQR)	16 (12–19)	20 (14–23.5)	15 (11–18)	<0.001	17 (13–21)	20 (17–24)	17 (13–21)	<0.001
SOFA, median (IQR)	7 (5–9)	9 (7–10)	6 (4–8)	<0.001	4 (3–6)	10 (7–11)	6 (5–9)	<0.001
AGI grade, No. (%)				<0.001				<0.001
I	516 (47.3%)	61 (27.6%)	455 (52.3%)		723 (80%)	68 (56.2%)	655 (83.7%)	
II	375 (34.4%)	77 (34.8%)	298 (34.3%)		143 (15.8%)	32 (26.4%)	111 (14.2%)	
III	145 (13.3%)	46 (20.8%)	99 (11.4%)		36 (4.0%)	19 (15.7%)	17 (2.2%)	
IV	55 (5.0%)	37 (16.7%)	18 (2.1%)		2 (0.2%)	2 (1.7%)	0	
GIDS				<0.001				<0.001
0	634 (58.1%)	63 (28.5%)	571 (65.6%)		744 (82.3%)	65 (53.7%)	679 (75.1%)	
1	293 (26.9%)	89 (40.3%)	204 (23.5%)		100 (11.1%)	18 (14.9%)	82 (10.5%)	
2	137 (12.5%)	57 (25.7%)	80 (9.2%)		49 (5.4%)	30 (24.8%)	19 (2.4%)	
3	26 (2.4%)	11 (5.0%)	15 (1.7%)		10 (1.1%)	7 (5.8%)	3 (0.4%)	
4	1 (0.1%)	1 (0.5%)	0 (0)		1 (0.1%)	1 (0.8%)	0 (0)	
mNUTRIC, median (IQR)	4 (3–5)	4 (3–6)	4 (3–5)	0.262	4 (3–6)	5 (3–6)	4 (3–5)	0.001
MV, No. (%)	829 (76.0%)	189 (85.5%)	640 (73.6%)	<0.001	614 (67.9%)	97 (80.2%)	517 (66.0%)	0.002
Maximum lactic acid, median (IQR), mmol/L	1.9 (1.3–2.8)	2 (1.5–3.3)	1.8 (1.3–2.8)	0.012	1.7 (1.1–2.6)	2 (1.4–3.4)	1.6 (1.1–2.5)	<0.001
28-day mortality, n (%)	221 (20.2%)	NA	NA	NA	121 (13.4%)	NA	NA	NA
LOS ICU	13 (8–20)	11 (7–18)	13 (8–22)	<0.001	11 (8–18)	13 (8–18)	11 (8–17)	0.195
ICU-free days to day 28	15 (6–20)	8 (0–19.5)	15 (6–20)	0.008	16 (6–20)	16 (9–20)	16 (5–20)	0.864

MV, mechanically ventilated; BMI, body mass index; AGI, acute gastrointestinal injury; GIDS, Gastrointestinal Dysfunction Score; APACHE II, Acute Physiology and chronic health evaluation II; SOFA, Sequential organ failure assessment; mNUTRIC, modified Nutrition Risk in the Critically Ill; IQR, interquartile range; SD, standard deviation; LOS, Length of Stay, NA, Not Applicable.

### 3.2 Comparison of the association of AGI and GIDS with 28-day mortality

#### 3.2.1 Overall distribution of AGI and GIDS grades and trends in 28-day mortality

**Figure 2:**
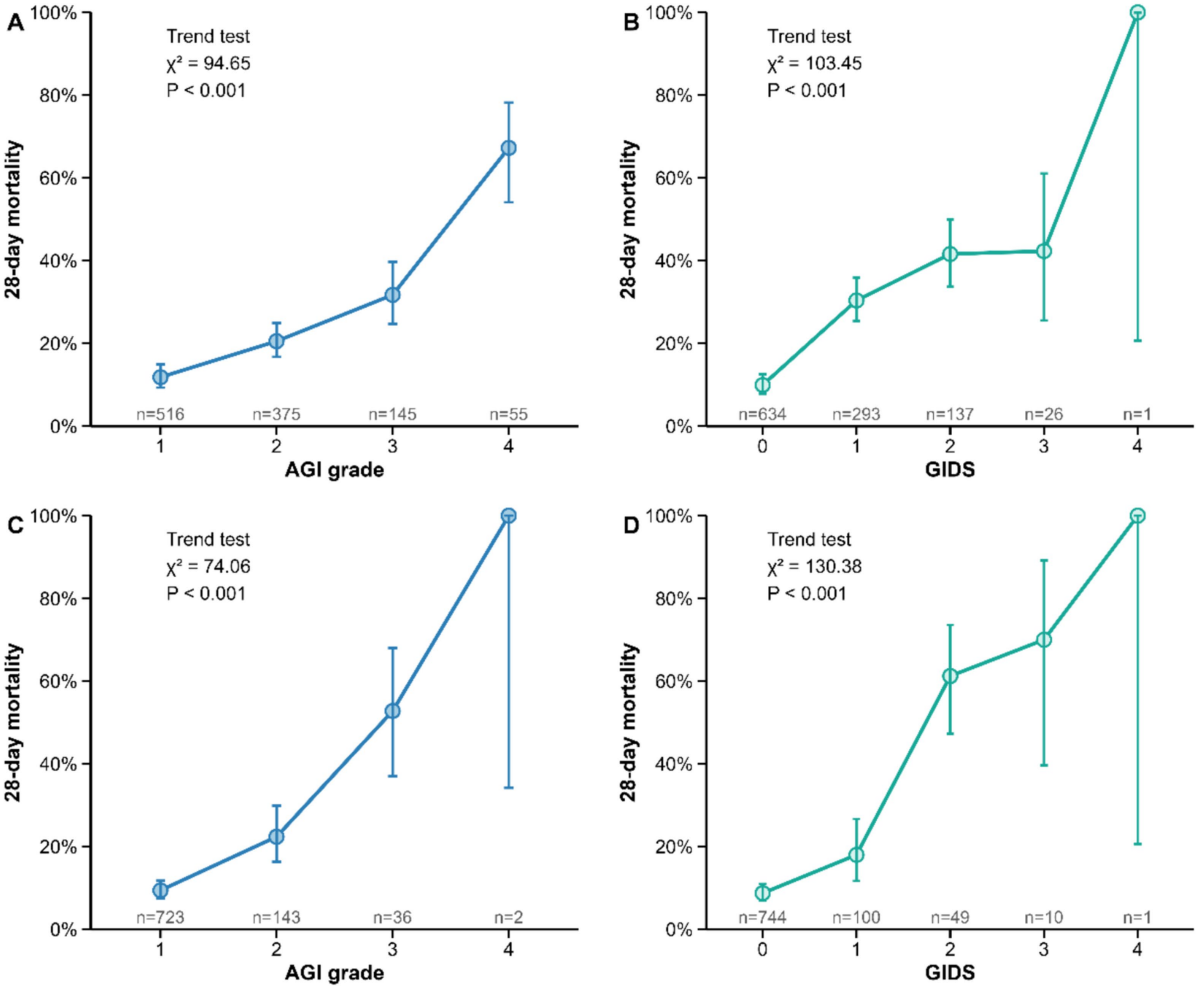
Distribution of patients and 28-day mortality according to AGI grades and GIDS categories. **(A,B)** FJLU cohort; **(C,D)** NEED trial dataset. AGI, acute gastrointestinal injury; GIDS, gastrointestinal dysfunction score; CIs, confidence intervals.

In the FJLU cohort, 28-day mortality increased with higher AGI grades and higher GIDS categories (Cochran–Armitage trend test, both *p* < 0.001), with a similar pattern observed in the NEED trial Dataset. The distributions of AGI grades and GIDS categories, along with the corresponding 28-day mortality with 95% CIs, are shown in Figure 2.

#### 3.2.2 Associations of AGI and GIDS with 28-day mortality

Univariable Cox regression analysis of AGI and GIDS for 28-day mortality:

In the FJLU cohort, both AGI grade and GIDS were positively associated with 28-day mortality. Nonetheless, in the joint models including AGI grade and GIDS, the difference between their effects did not reach statistical significance (Wald test:z = 0.27, *p* = 0.785). The NEED Trial yielded similar results (Supplementary Table S4).

Multivariable Cox models for the associations of AGI and GIDS with 28-day mortality:

Detailed results of the univariable Cox analyses and collinearity assessment of the candidate variables are shown in Supplementary Tables S5, S6.

In the FJLU cohort, higher AGI and GIDS scores were consistently associated with an increased risk of 28-day mortality in models adjusted for potential confounders (Supplementary Table S7). When both scores were entered simultaneously, AGI and GIDS showed very similar adjusted effects on mortality (HR per 1-point increase approximately 1.45 vs. 1.49; identical HR 1.38 per 1-SD increase) (Table 2). In this joint model, Wald tests showed no significant difference in the per-point log-hazard ratios for AGI and GIDS (Wald test:z = 0.18, *p* = 0.857). Global Schoenfeld tests did not indicate violations of the proportional hazards assumption for either score in the FJLU cohort (*p* = 0.083). Analyses in the NEED trial cohort demonstrated a similar pattern of associations and model diagnostics (Table 2; Supplementary Table S7).

**Table 2:** Joint multivariable Cox models for the associations of AGI and GIDS with 28-day mortality in the FJLU and NEED cohorts.

Variable	Scale	FJLU Cohort		NEED Trial	
		HR (95% CI)	*p* value	HR (95% CI)	*p* value
AGI	Per 1-point	1.45 (1.24–1.70)	<0.001	1.24 (0.91–1.71)	0.175
	Per 1 SD	1.38 (1.20–1.58)	<0.001	1.12 (0.95–1.33)	0.175
GIDS	Per 1-point	1.49 (1.27–1.75)	<0.001	1.85 (1.45–2.37)	<0.001
	Per 1 SD	1.38 (1.21–1.57)	<0.001	1.46 (1.26–1.70)	<0.001

Data are from covariate-adjusted joint Cox models including both AGI and GIDS. Hazard ratios are presented per 1-point increase and per 1-standard deviation increase.

AGI, acute gastrointestinal injury; GIDS, Gastrointestinal Dysfunction Score; SD, standard deviation. HR, Hazard ratio; CI, confidence interval.

#### 3.2.3 Discriminative performance of AGI grade and GIDS for 28-day mortality

##### 3.2.3.1 Overall discriminative performance of AGI grade and GIDS for 28-day mortality

**Figure 3:**
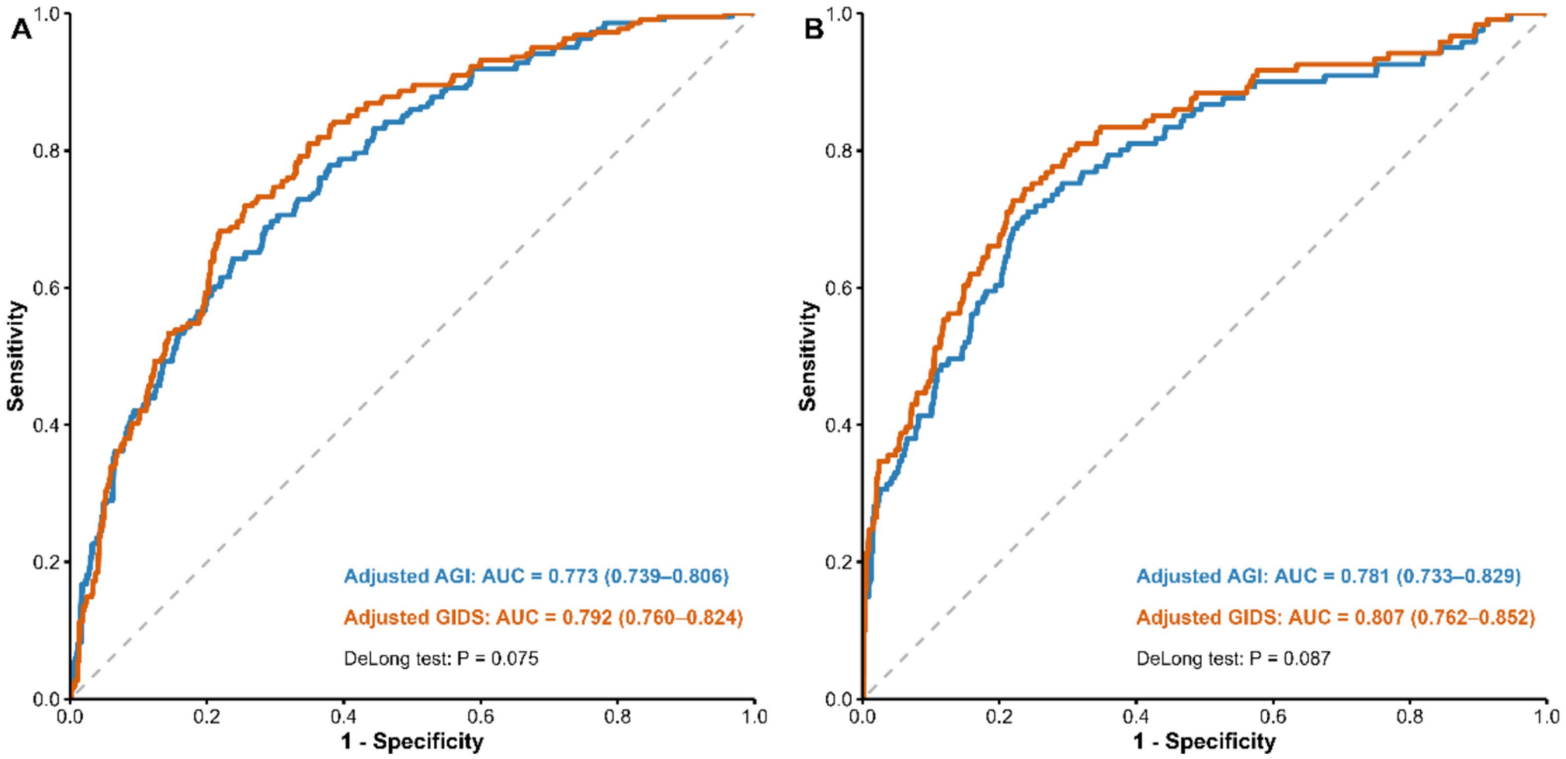
Comparison of confounder-adjusted ROC of adjusted AGI grade and GIDS for 28-day mortality in the two cohorts: **(A)** FJLU cohort and **(B)** NEED cohort. AGI, acute gastrointestinal injury; GIDS, gastrointestinal dysfunction score; ROC, receiver operating characteristic curve; AUC, area under the curve; adjusted AGI: multivariable model including AGI grade, adjusted for potential confounders; adjusted GIDS: multivariable model including GIDS, adjusted for potential confounders.

Across both cohorts, GIDS yielded numerically higher AUCs for 28-day mortality than AGI grade, although the differences were not statistically significant in either the unadjusted analyses (Supplementary Figures S1A,B) or the adjusted analyses (Figure 3) (all *p* > 0.05 by DeLong’s test). Calibration was also comparable between the two scores in both cohorts, as shown by the calibration curves and corresponding statistical analyses in the unadjusted setting (Supplementary Figures S1C, D and Supplementary Table S8) and after covariate adjustment (Supplementary Figure S2 and Supplementary Table S9).

##### 3.2.3.2 Subgroup analyses of the discriminative performance of AGI grade and GIDS

**Figure 4:**
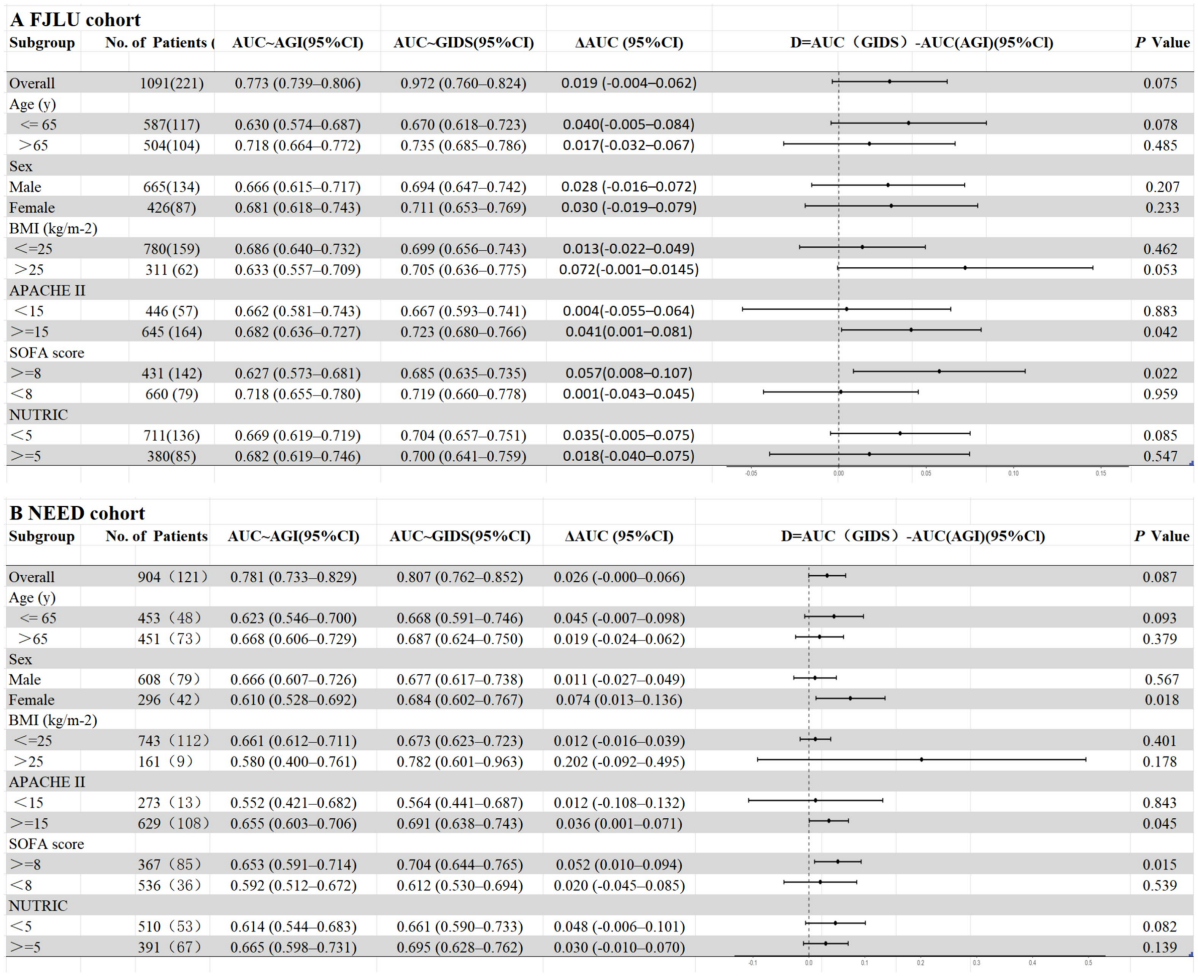
Subgroup analyses of discrimination performance for predicting 28-day mortality in the two cohorts. **(A)** FJLU cohort. **(B)** NEED cohort. Differences in AUC (ΔAUC) between GIDS and AGI were assessed using the DeLong test. BMI, body mass index; AGI, acute gastrointestinal injury; GIDS, Gastrointestinal Dysfunction Score; APACHE II, Acute physiology and chronic health evaluation II; SOFA, Sequential rgan ailure ssessment; mNUTRIC, modified Nutrition Risk in the Critically Ill; AUC, area under the receiver operating characteristic curve; CI, confidence interval.

Subgroup analyses for 28-day mortality discrimination demonstrated that GIDS generally produced numerically higher AUCs than AGI in both datasets (Supplementary Figures S3, S4); however, these differences were not statistically significant in most subgroups. In the FJLU cohort, statistically significant improvements were observed only in patients with APACHE II > 15 (ΔAUC = 0.041, 95% CI 0.001–0.081; *p* = 0.042) and SOFA score ≥8 (ΔAUC = 0.057, 95% CI 0.008–0.107; *p* = 0.022) (Figure 4A). Similar results were observed in the NEED cohort (Figure 4B). Detailed subgroup ROC analyses are shown in Supplementary Figures S5, S6 for the FJLU and NEED cohorts, respectively. In the subgroups with APACHE II ≥ 15 and SOFA score ≥8, confounder-adjusted ROC curves continued to show better discrimination for GIDS than for AGI grade in both cohorts (Supplementary Figure S7). Calibration after confounder adjustment remained comparable between the two scores in these subgroups, as shown by the calibration curves (Supplementary Figure S8) and corresponding statistical analyses (Supplementary Tables S10, S11).

### 3.3 Comparison of the associations of AGI grade and GIDS with ICU-free days to day 28

**Figure 5:**
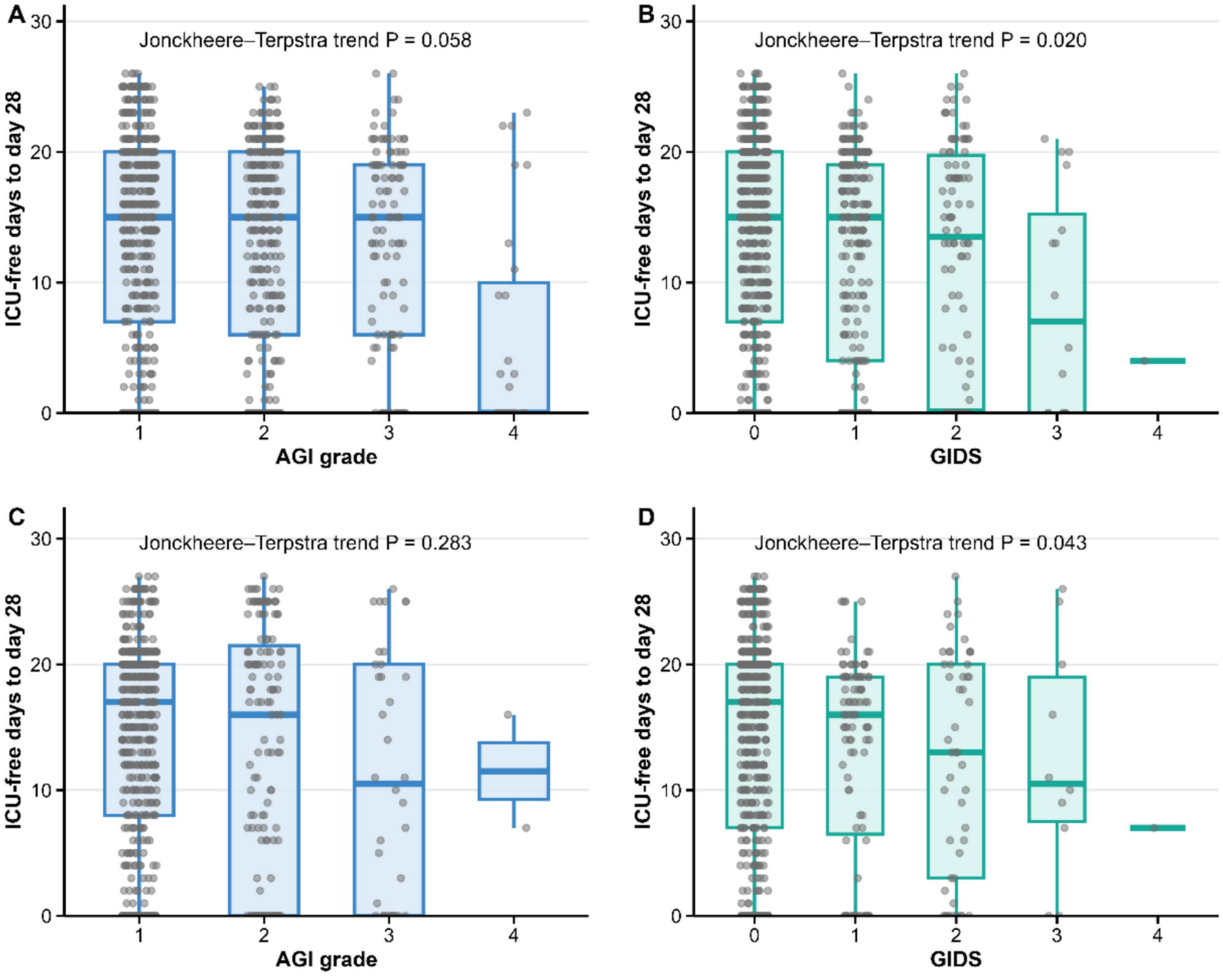
Distribution of ICU-free days to day 28 across AGI grade and GIDS categories in the two cohorts. **(A,B)** FJLU cohort; **(C,D)** NEED trial cohort. GIDS, Gastrointestinal Dysfunction Score; AGI, acute gastrointestinal injury; CI, confidence interval.

For the secondary outcome of ICU-free days to day 28, the primary trend analyses suggested a more consistent inverse association for GIDS across both cohorts (Figure 5). Supplementary covariate-adjusted linear regression analyses with robust standard errors showed broadly concordant results, although joint models including both AGI grade and GIDS did not demonstrate clear superiority of either score (Supplementary Table S12). Residual diagnostics for the linear models are shown in Supplementary Figure S9.

## 4 Discussion

In this study, we compared the associations of AGI grade and the GIDS with key short-term clinical outcomes in two large cohorts of critically ill patients with baseline gastrointestinal injury. We found that both AGI grade and GIDS were significantly associated with 28-day mortality, while GIDS showed additional discriminatory value in patients with greater illness severity. For the secondary outcome of ICU-free days to day 28, GIDS showed a more consistent adverse association than AGI grade across both cohorts. This finding suggests that GIDS may better capture the bedside burden of worsening gastrointestinal dysfunction during ICU stay, which may be relevant for ongoing monitoring, nutritional progression, and multidisciplinary care planning. To our knowledge, this is the first large-sample study to compare the clinical relevance of GIDS and AGI using two independent cohorts to improve the robustness and reproducibility of the findings.

In contrast to Shen et al. (19), who found that GIDS was an unreliable predictor of 28-day mortality, we observed a clear stepwise increase in 28-day mortality across increasing AGI grades and GIDS categories. This graded pattern was supported by trend analyses and is consistent with previous studies showing higher mortality with worsening gastrointestinal dysfunction in critically ill patients (6, 24–27). In Cox regression analyses treating both scores as ordinal variables, each 1-point increase in AGI grade or GIDS category was associated with an approximately two- to threefold higher hazard of 28-day mortality. However, when AGI grade and GIDS were entered simultaneously into the same Cox model, their regression coefficients did not differ significantly on Wald testing, suggesting that their prognostic associations were broadly comparable. One possible explanation is that, although GIDS may capture more dimensions of gastrointestinal dysfunction than AGI, the incremental prognostic information it provides may be limited. AGI grade itself already reflects major manifestations of gastrointestinal dysfunction and overall disease severity, which may reduce the additional discriminatory gain achievable with a more complex scoring system in the overall ICU population. In addition, some GIDS components may overlap with broader indicators of critical illness severity, potentially limiting their independent prognostic contribution. However, the potential advantages of GIDS may become more apparent in patients with greater illness severity, which is consistent with our subgroup analyses showing improved discrimination in patients with higher APACHE II and SOFA scores.

Among patients with higher disease severity (APACHE II ≥ 15 or SOFA ≥8), GIDS appeared to provide better discrimination than AGI grade for 28-day mortality, while calibration performance remained comparable, and these differences persisted after multivariable adjustment. Several factors may explain these differences. First, in patients with more severe conditions, such as those experiencing shock, systemic inflammatory responses, microcirculatory disturbances, gastrointestinal dysmotility, and barrier disruption, multiple pathways of gastrointestinal dysfunction often occur simultaneously (3, 4). GIDS may more comprehensively capture the multidimensional nature of gastrointestinal dysfunction. Second, under critical illness, the effects of sedatives, opioids, vasoactive agents, and nutritional strategies on gastrointestinal function are further amplified. The more complex structure of the GIDS may better capture these graded influences, allowing a more accurate and nuanced assessment of gastrointestinal dysfunction severity. These findings suggest that, in patients with greater illness severity, GIDS may provide additional discriminatory value beyond AGI grade for assessing the prognostic impact of gastrointestinal dysfunction.

To more comprehensively compare the clinical relevance of GIDS and AGI grade, we also evaluated ICU-free days to day 28 as a secondary outcome. This endpoint is a composite measure that reflects survival status, duration of critical illness, and ICU resource utilization, and as a quasi-continuous variable it may be more sensitive than mortality alone for identifying differences in risk stratification performance between scoring systems. For this secondary outcome, GIDS showed a more consistent adverse association than AGI grade across both cohorts. In the primary trend analyses, higher GIDS categories were associated with progressively fewer ICU-free days, whereas the corresponding trend for AGI grade was weaker and not consistently statistically significant. This pattern suggests that GIDS may better reflect the short-term clinical burden of gastrointestinal dysfunction during ICU stay. However, the supplementary covariate-adjusted linear regression analyses were less conclusive: although directionally consistent, joint models including both AGI grade and GIDS did not demonstrate clear superiority of either score. Accordingly, these findings should be interpreted as supportive, rather than definitive, evidence of greater clinical utility for GIDS over AGI grade with respect to ICU-free days to day 28.

Beyond its prognostic utility, our findings may also have implications for supportive care in the ICU. Although our study focused on short-term prognostic performance rather than nutrition-related endpoints, the findings may still be relevant to ICU nutritional practice, as gastrointestinal dysfunction frequently compromises enteral feeding tolerance and adequate nutrient delivery. In this context, a structured gastrointestinal assessment tool may help identify patients who require closer monitoring and more individualized nutritional support.

This study has several strengths. First, our findings were replicated in two independent datasets, including a nationwide, multicenter, cluster-randomized clinical trial cohort, supporting the robustness and reproducibility of the findings across two independent cohorts. Second, AGI grade and GIDS were scored by independent assessors, which minimized potential cross-contamination between the two scoring systems. In addition, we focused on two key clinical outcomes, which further enhance the clinical applicability of our conclusions. This study also has several limitations. First, although we performed multivariable adjustment, the observational nature of the analysis does not allow complete elimination of residual confounding. Second, the number of patients with AGI grade 4 and GIDS 4 was relatively small in both datasets, which widened the CIs and increased the risk of model instability in these subgroups. Third, in the NEED cohort, GIDS was retrospectively reconstructed from previously collected clinical records rather than prospectively assessed at the bedside. Although predefined criteria were used for score derivation, some gastrointestinal variables were unavailable in a subset of patients, and GIDS had to be derived from the remaining available components. Therefore, some degree of information bias cannot be completely excluded. In addition, patients with incomplete data were excluded from the validation analysis. Because the missingness may not have been completely random, this complete-case analysis may have introduced selection bias and affected the representativeness and generalizability of the validation cohort. A small number of patients in the primary cohort were also lost to follow-up, which may have influenced outcome assessment. Consequently, the study findings should be interpreted with caution. In addition, both cohorts were derived from Chinese ICU populations, including one single-center cohort and one nationwide multicenter cohort. Differences in ethnicity, healthcare systems, ICU management practices, nutritional strategies, and gastrointestinal assessment approaches may limit the generalizability of these findings to other regions and healthcare settings. Future studies should include prospective, international multicenter cohorts to further validate the performance and clinical utility of GIDS across different healthcare systems and ICU populations. In addition, whether GIDS-guided gastrointestinal management strategies can improve patient outcomes warrants further investigation.

Overall, our findings suggest that GIDS may have practical value for bedside monitoring and care planning in critically ill patients. However, as its apparent advantage over AGI was not uniformly confirmed across all analyses, further validation is needed before routine implementation can be recommended.

## 5 Conclusion

Both GIDS and AGI were associated with short-term outcomes in critically ill patients. Although their overall prognostic performance was comparable, GIDS may offer incremental clinical utility in more severely ill patients and may better capture the short-term consequences of gastrointestinal dysfunction. These findings should be interpreted cautiously and require further validation.

## Data Availability

The original contributions presented in the study are included in the article/Supplementary material, further inquiries can be directed to the corresponding authors.
