# Supplementary material for: Clinical utility of the gastrointestinal dysfunction score versus acute gastrointestinal injury grade for short-term risk stratification in critically ill patients: evidence from two prospective cohorts
Source: Front Nutr. 2026 Jun 10;13:1870956. doi: 10.3389/fnut.2026.1870956 (PMC13292638; doi:10.3389/fnut.2026.1870956)
Supplement: Supplementary file 1 [file Data_Sheet_1.docx]

Clinical utility of the Gastrointestinal Dysfunction Score versus Acute Gastrointestinal Injury grade for short-term risk stratification in critically ill patients: evidence from two prospective cohorts

Yanhua Li ^1^, Youquan Wang ^1^, Yuhan Zhang ^1^, Feng Zhang ^1^, Lu Ke ^2,3*^, Dong Zhang ^1*^ and for the Chinese Critical Care Nutrition Trials Group (CCCNTG)

^1^ Department of Critical Care Medicine, The First Hospital of Jilin University, Changchun, 130021, China

^2^ Department of Critical Care Medicine, Jinling Hospital, Medical School of Nanjing University, Nanjing 210002, Jiangsu, China; ^3^ National Institute of Healthcare Data Science, Nanjing University, Nanjing 210010, Jiangsu, China

*Corresponding Author:

Lu Ke email: [kelu@nju.edu.cn](mailto:kelu@nju.edu.cn)

**Corresponding Author:

Dong Zhang, email: [zhangdong@jlu.edu.cn](mailto:zhangdong@jlu.edu.cn)

Additional emails:

Yanhua Li email: [liyanhua@jlu.edu.cn](mailto:liyanhua@jlu.edu.cn)

Youquan Wang email: [wangyq20@mails.jlu.edu.cn](mailto:wangyq20@mails.jlu.edu.cn)

Yuhan Zhang email: zhangyuhan23@jlu.edu.cn

Feng Zhang email: [zhangfengmodest@163.com](mailto:zhangfengmodest@163.com)

**Contents of the supplementary appendix:**

1. Assessment of Gastrointestinal Dysfunction Severity………………………………….………4

Supplemental Table S1 Classification of AGI Grade…………………………………………...4

Supplemental Table S2 Gastrointestinal Dysfunction Score (GIDS)………………………...... 5

Supplemental Table S3 Definition of variables in Gastrointestinal Dysfunction Score (GIDS).6

1. Comparison of the associations of AGI and GIDS with 28-day mortality....................................7
   1. Univariable Cox regression analysis of AGI and GIDS for 28-day mortality.......................7

Supplementary Table S4. Association of AGI grade and GIDS with 28-day mortality in univariable Cox regression..........................................................................................................7

- 1. Multivariable-adjusted Cox regression analysis of AGI and GIDS for 28-day mortality....7

Supplemental Table S5 Univariable Cox regression analysis of candidate variables for 28-day mortality in the two cohorts .......................................................................................................8

Supplementary Table S6 Variance inflation factors for covariates in multivariable Cox models in the two cohorts........................................................................................................................9

Supplemental Table S7 Association of AGI grade and GIDS with 28-day mortality in Multivariate Cox regression analysis in the two cohorts............................................................10

- 1. Discriminative performance of AGI grade and GIDS for 28-day mortality........................11

2.3.1 Overall Discriminative performance of AGI grade and GIDS for 28-day mortality

Supplemental Table S8 Statistical Analysis of Calibration for AGI and GIDS predicting 28-day Mortality in two datasets...........................................................................................................11

Supplementary Figure S1. ROC curves and calibration plots of AGI grade and GIDS for predicting 28-day mortality in two cohorts...............................................................................11

Supplementary Table S9 Statistical Analysis of Calibration Curves for the two cohorts after Adjustment for Confounding factors........................................................................................12

Supplementary Figure S2. Confounder-adjusted calibration curves of AGI grade and GIDS for predicting 28-day mortality in two cohorts...............................................................................12
2.3.2 Subgroup analyses of the discriminative performance of AGI grade and GIDS.............12

Supplementary Figure S3 Forest plot of AUC for 28-day mortality of AGI and GIDS in each subgroup of the FJLU Cohort Dataset.......................................................................................13

Supplementary Figure S4 Forest plot of AUC for 28-day mortality of AGI and GIDS in each subgroup of the NEED Trial Dataset.........................................................................................14

Supplementary Figure S5. Subgroup ROC curves comparing AGI grade and GIDS for predicting 28-day mortality in the FJLU cohort........................................................................15

Supplementary Figure S6. Subgroup ROC curves comparing AGI grade and GIDS for predicting 28-day mortality in the NEED cohort......................................................................16

Supplementary Figure S7. Confounder-adjusted ROC curves comparing AGI grade and GIDS for predicting 28-day mortality in patients with APACHE II ≥15 and SOFA ≥8 in two cohorts......................................................................................................................................17

Supplementary Figure S8. Confounder-adjusted calibration curves of AGI grade and GIDS for predicting 28-day mortality in patients with APACHE II ≥15 and SOFA ≥8 in two cohorts .. 18

Supplementary Table S10. Confounder-adjusted calibration of AGI grade and GIDS for 28-day mortality in patients with APACHE II ≥15 in two cohorts........................................................19

Supplementary Table S11. Confounder-adjusted calibration of AGI grade and GIDS for 28-day mortality in patients with SOFA ≥8 in two cohorts...................................................................19

1. Comparison of the Associations of AGI Grade and GIDS with ICU-Free Days at Day 28 .... 20

Supplementary Figure S9. Residual diagnostics for the linear regression model including AGI grade, GIDS, covariates, and ICU-free days at day 28.............................................................20

Supplementary Table S12. Multivariable linear regression of AGI and GIDS in relation to ICU-free days to day 28 with robust standard errors.........................................................................21

1. References.................................................................................................................................21

**1.** **Assessment of Gastrointestinal Dysfunction Severity**

**Supplemental Table S1(1) Classification of AGI Grade**

| Grade | Definition |
| --- | --- |
| I (risk of GI dysfunction or failure) | Partial impairment of GI function, manifested as gastrointestinal symptoms related to a known cause and perceived to be transient. Examples: postoperative nausea and/or vomiting during the first few days after abdominal surgery, postoperative absence of bowel sounds, diminished bowel motility in the early phase of shock. |
| II (GI dysfunction) | The GI tract is unable to perform digestion and absorption adequately to satisfy the nutrient and fluid requirements of the body. There are no changes in the general condition of the patient due to GI problems. Examples: gastroparesis with high gastric residuals or reflux, paralysis of the lower GI tract, diarrhea, intra-abdominal pressure (IAP) 12–15 mmHg, visible blood in gastric content or stool. Feeding intolerance is present if at least 20 kcal/kg BW/day via the enteral route cannot be achieved within 72 h of a feeding attempt. |
| III (GI failure) | Loss of GI function. Restoration of GI function is not achieved despite interventions, and the general condition is not improving. Examples: persistent feeding intolerance despite treatment manifested as high gastric residuals, persistent GI paralysis, occurrence or worsening of bowel dilatation, IAP, 15–20 mmHg, low abdominal perfusion pressure (below 60 mmHg). Feeding intolerance is present and possibly associated with persistence or worsening of multiple organ dysfunction syndrome. |
| IV (GI failure with severe impact on distant organ function) | AGI has progressed to become directly and immediately life-threatening, with worsening of multiple organ dysfunction syndrome and shock. Examples: bowel ischemia with necrosis, GI bleeding leading to hemorrhagic shock, Ogilvie syndrome, abdominal compartment syndrome requiring decompression. |

AGI acute gastrointestinal injury, BW body weight, GI gastrointestinal, IAP intra-abdominal pressure

**Supplemental Table S2. Gastrointestinal Dysfunction Score (GIDS)**

| 0 (No risk) | 1 (Increased risk) | 2 (GI dysfunction) | 3 (GI failure) | 4 (Life-threatening) |
| --- | --- | --- | --- | --- |
| No symptoms OR one of the following with oral intake | Two of the following: | Three or more symptoms of score 1 OR up to two of the following: | Three or more of the following: | One of the following: |
| • Absent bowel sounds • Vomiting • GRV > 200 mL (single measurement) • GI paralysis/dynamic ileus • Abdominal distension • Diarrhea (not severe) • GI bleeding without transfusion • IAP 12–20 mmHg | • No oral intake • Absent bowel sounds • Vomiting • GRV > 200 mL (single measurement) • GI paralysis/dynamic ileus • Abdominal distension • Diarrhea (not severe) • GI bleeding without transfusion • IAP 12–20 mmHg | • Severe diarrhea • GI bleeding with transfusion • IAP > 20 mmHg | • Prokinetic use • GI paralysis/dynamic ileus • Abdominal distension • Severe diarrhea • GI bleeding with transfusion • IAP > 20 mmHg | GI bleeding leading to hemorrhagic shock • Mesenteric ischemia • Abdominal compartment syndrome (ACS) |

*Notes: If certain variables (e.g., GRV, IAP) have not been measured, the score can still be calculated by excluding these variables.
Abbreviations: GI gastrointestinal, GRV, gastric residual volume; IAP, intra-abdominal pressure; ACS, abdominal compartment syndrome.*

**Supplemental Table S3 Definition(1) of variables in Gastrointestinal Dysfunction Score (GIDS)**

| Variable | Definition |
| --- | --- |
| Absent bowel sounds | Yes/no. We advised to confirm absence of bowel sound in at least 2 assessments both in at least 2 quadrants. |
| Vomiting/regurgitation | Yes/no. The occurrence of any visible regurgitation of gastric content irrespective of the amount. |
| Maximum gastric residual volume (GRV) | Per one measurement and total per day. The following details of GRV measurement were recorded: number of measurements/24h, maximum GRV per one measurement, tube size, active vs passive measurement, aspirated volume returned vs discarded. |
| Stool details | Times per day for each category of the Bristol stool chart were recorded, or the amount in mL in case a stool collector was used. Diarrhea was defined as having three or more loose (Bristol scale 5 or higher) or liquid stools per day with a stool amount greater than 250 mL/day. Severe diarrhea was defined as stools with Bristol scale 6-7 for ≥5 times or ≥1000 mL with stool collector on this day. |
| Abdominal distension | Yes/no. Based on subjective evaluation. |
| Bowel distension | Yes/no. Diagnosed ultrasound with details on location (small or large bowel) and bowel diameter (cm). |
| GI bleeding | Yes/no. An amount of visible blood in vomit, gastric aspirate or stool with the following details: upper/lower, and massive bleeding leading to transfusion: yes/no. |
| GI paralysis/dynamic ileus | Yes/no. Based on clinical assessment, no stool ≥3 consecutive days. |
| Bowel obstruction | Yes/no. Based on any investigations leading to respective diagnosis. |
| Intraabdominal pressures | IAP measurements were performed at least once in every 6-12 hrs after a urinary bladder catheter was placed for standard treatment purposes. Mean arterial pressure (MAP) was documented simultaneously with each IAP measurement for calculation of abdominal perfusion pressure (APP) (APP=MAP-IAP). |
| Intra-abdominal hypertension | Defined as sustained IAP ≥12mmHg – was considered present when mean IAP of all performed measurements on one study day was ≥12 mmHg. |
| Abdominal Compartment Syndrome | Defined as sustained IAP >20mmHg with new organ dysfunction/failure and documented as yes/no for each study day. |

*GIDS, Gastrointestinal Dysfunction Score; GRV, gastric residual volume; GI, gastrointestinal; IAP, intra-abdominal pressure; MAP, mean arterial pressure; APP, abdominal perfusion pressure.*

**2. Comparison of the associations of AGI and GIDS with 28-day mortality**

2.1 Univariable Cox regression analysis of AGI and GIDS for 28-day mortality

In both the FJLU cohort and the NEED trial, higher AGI and GIDS scores were strongly associated with increased 28-day mortality in univariable Cox models (*Supplemental Table S4*). In joint models including both scores, AGI and GIDS remained independently associated with mortality in the FJLU cohort, whereas in the NEED trial the association for AGI was weaker and GIDS showed a clearly stronger effect; after standardization, per-SD effects were similar in FJLU but larger for GIDS than for AGI in NEED. Gobal Schoenfeld tests indicated no relevant violation of the proportional hazards assumption (FJLU: *P* = 0.184; NEED: *P* = 0.131).

**Supplementary Table S4. Association of AGI grade and GIDS with 28-day mortality in univariable Cox regression**

| Model | variable | FJLU Cohort | | NEED Trial | |
| --- | --- | --- | --- | --- | --- |
|  |  | HR (95% CI) | P value | HR (95% CI) | P value |
| Univariable Cox | AGI | 1.90 (1.67–2.17) | ＜0.001 | 2.51 (2.14–2.95) | ＜0.001 |
|  | GIDS | 1.94 (1.71–2.21) | ＜0.001 | 2.62 (2.10–3.26) | ＜0.001 |
| Joint Cox model  (AGI and GIDS entered simultaneously) | AGI | 1.49 (1.28–1.75) | ＜0.001 | 1.37 (1.00–1.86) | 0.048 |
|  | GIDS | 1.55 (1.32–1.82) | ＜0.001 | 2.12 (1.68–2.69) | ＜0.001 |
| Joint Cox model with standardized scores | AGI_z | 1.42 (1.24–1.62) | ＜0.001 | 1.18 (1.00–1.39) | 0.048 |
|  | GIDS_z | 1.42 (1.25–1.62) | ＜0.001 | 1.59 (1.37–1.84) | ＜0.001 |

*AGI, acute gastrointestinal injury; GIDS, Gastrointestinal Dysfunction Score;* *HR, Hazard ratio; CI, confidence interval; AGI_z,z-standardized AGI grade (per 1-SD increase), GIDS_z, z-standardized GIDS grade (per 1-SD increase)*

2.2 Multivariable-adjusted Cox regression analysis of AGI and GIDS for 28-day mortality

To address potential confounding, we conducted multivariable Cox proportional hazards analyses. Candidate covariates were prespecified as age, sex, BMI, primary diagnosis, APACHE II score, SOFA score, AGI grade, GIDS, receipt of mechanical ventilation, and the highest lactate within the first 24 hours. We first performed univariable Cox analyses in both datasets (*Supplemental Table S5*).

In the FJLU Cohort Dataset, variables with p > 0.10 (sex, BMI, NUTRIC score, and diagnosis) were excluded. APACHE II was omitted because of collinearity with age and SOFA. Given the potential collinearity between AGI grade and GIDS, each was entered into separate multivariable models to mitigate covariance (*details of collinearity testing in Supplemental Table S6*). In the final multivariable models, age, SOFA score, gastrointestinal dysfunction score, and mechanical ventilation emerged as independent predictors of 28-day mortality *(Supplemental Table S7*).

In the NEED Trial Dataset, variables with p > 0.10 on univariable screening were likewise excluded. APACHE II and NUTRIC were additionally removed due to collinearity with age and SOFA. As above, AGI grade and GIDS were modeled separately to address collinearity (*Supplemental Table S6*). The multivariable results identified age, BMI, gastrointestinal dysfunction score, SOFA score, and mechanical ventilation as independent risk factors for 28-day mortality (*Supplemental Table S7*).

**Supplemental** **Table S5** **Univariable Cox regression analysis of candidate variables for 28-day mortality in the two Cohorts**

| Variables | FJLU Cohort Dataset | | NEED Trial Dataset | |
| --- | --- | --- | --- | --- |
|  | HR (95%CI) | P value | HR (95%CI) | P value |
| Age | 1.01（1.0-1.02） | 0.049 | 1.01(1.00-1.03) | 0.022 |
| Sex | 1.02（0.750-1.37） | 0.913 | 0.903(0.607-1.36) | 0.620 |
| BMI | 0.97（1.01-1.10） | 0.110 | 0.859(0.803-0.92) | ＜0.001 |
| Apache II | 1.18（1.21-2.11） | ＜0.001 | 1.13（1.09-1.17） | ＜0.001 |
| SOFA | 1.25（1.19-1.31） | ＜0.001 | 1.29（1.21-1.38） | ＜0.001 |
| mNUTRIC | 1.05（0.961-1.15） | 0.279 | 1.18（1.07-1.30） | 0.001 |
| AGI grade | 1.90 (1.67–2.17) | ＜0.001 | 2.62 (2.10–3.26) | ＜0.001 |
| GIDS | 1.94 (1.71–2.21) | ＜0.001 | 2.51 (2.14–2.95) | ＜0.001 |
| Maximum lactic within 24 h | 1.10（1.02-1.18） | 0.012 | 1.05（0.990-1.10） | 0.085 |
| Mechanical ventilation | 2.12(1.44-3.23) | ＜0.001 | 2.08（1.32-3.40） | 0.002 |
| Diagnosis | 0.99（0.897-1.11） | 0.981 | 0.71（0.594-0.84） | ＜0.001 |

*APACHE II, Acute Physiology and Chronic Health Evaluation II; SOFA, Sequential Organ Failure Assessment*; *BMI, Body Mass Index; mNUTRIC, modified Nutrition Risk in the Critically Ill,AGI, acute gastrointestinal injury; GIDS, Gastrointestinal Dysfunction Score; HR, Hazard ratio; CI, confidence interval*

**Supplementary Table S6 Variance inflation factors for covariates in multivariable Cox models in the two cohorts**

| Variables | FJLU Cohort Dataset | | | | NEED Trial Dataset | | | |
| --- | --- | --- | --- | --- | --- | --- | --- | --- |
|  | Model 1 (AGI grade) | | Model 2 (GIDS) | | Model 1 (AGI grade) | | Model 2 (GIDS) | |
|  | VIF | \| Tolerance \| \| --- \| | VIF | Tolerance | VIF | Tolerance | VIF | Tolerance |
| Age | 1.011 | 0.989 | 1.010 | 0.990 | 1.023 | 0.977 | 1.019 | 0.981 |
| EGI | 1.004 | 0.996 | 1.005 | 0.995 | 1.042 | 0.960 | 1.011 | 0.989 |
| Mechanical ventilation | 1.026 | 0.974 | 1.022 | 0.978 | 1.020 | 0.980 | 1.025 | 0.975 |
| SOFA | 1.024 | 0.976 | 1.029 | 0.972 | 1.032 | 0.969 | 1.021 | 0.980 |
| BMI |  |  |  |  | 1.042 | 0.959 | 1.029 | 0.972 |
| Maximum lactic within 24 h | 1.012 | 0.987 | 1.011 | 0.988 | 1.015 | 0.985 | 1.021 | 0.980 |

*EGI, Evaluation of Gastrointestinal Injury; SOFA, Sequential Organ Failure Assessment*; *BMI, Body Mass Index; AGI, acute gastrointestinal injury; GIDS, Gastrointestinal Dysfunction Score; VIF, Variance Inflation Factor*

**Supplemental Table S7 Association of AGI grade and GIDS with 28-day mortality in Multivariate Cox regression analysis in the two cohorts**

| Variables | FJLU Cohort | | | | NEED Trial | | | |
| --- | --- | --- | --- | --- | --- | --- | --- | --- |
|  | Model 1 (AGI grade) | | Model 2 (GIDS) | | Model 1 (AGI grade) | | Model 2 (GIDS) | |
|  | HR (95%CI) | P | HR (95%CI) | P | HR (95%CI) | P | HR (95%CI) | P |
| Age | 1.01（1.00-1.02） | 0.041 | 1.01（1.00-1.02） | 0.041 | 1.01（1.00-1.03） | 0.038 | 1.01（1.00-1.03） | 0.073 |
| SOFA | 1.21（1.15-1.27） | ＜0.001 | 1.22（1.16-1.29） | ＜0.001 | 1.22（1.14-1.32） | ＜0.001 | 1.22（1.14-1.32） | ＜0.001 |
| EGI | 2.10（1.76-2.51） | ＜0.001 | 2.42（2.00-2.94） | ＜0.001 | 2.92（2.09-4.10） | ＜0.001 | 3.01（2.29-4.00） | ＜0.001 |
| BMI | NA | NA | NA | NA | 0.843(0.783-0.906) | ＜0.001 | 0.862(0.799-0.982) | ＜0.001 |
| Maximum lactic within 24 h | 1.07（0.983-1.16） | 0.112 | 1.06（0.977-1.15） | 0.155 | 1.04（0.976-1.10） | 0.142 | 1.03（0.967-1.09） | 0.262 |
| Mechanical ventilation | 1.68(1.10-2.64) | 0.020 | 1.61 (1.05-2.53) | 0.031 | 1.68(1.02-2.85) | 0.046 | 1.62 (0.969-2.81) | 0.073 |

*AGI, acute gastrointestinal injury; GIDS, Gastrointestinal Dysfunction Score; SOFA, Sequential organ failure assessment; EGI, Evaluation of Gastrointestinal Injury;*

*HR, Hazard ratio; CI, confidence interval; NA,* *not applicable.*

**2.3** Discriminative performance of AGI grade and GIDS for 28-day mortality

2.3.1 Overall Discriminative performance of AGI grade and GIDS for 28-day mortality

**Supplemental Table S8 Statistical Analysis of Calibration for AGI and GIDS predicting 28-day Mortality in two datasets**

|  | FJLU Cohort Dataset | | | | | | NEED Trial Dataset | | | | | |
| --- | --- | --- | --- | --- | --- | --- | --- | --- | --- | --- | --- | --- |
|  | intercept | p | Slope | p | ΔICI  (GIDS-AGI) | p | intercept | p | Slope | p | ΔICI  (GIDS-AGI) | p |
| AGI grade | 0 | 1 | 1 | 1 | 0.006  （-0.032-0.020） | 0.65 | 0 | 1 | 1 | 1 | 0.004  （-0.019, 0.010） | 0.52 |
| GIDS | 0 | 1 | 1 | 1 |  |  | 0 | 1 | 1 | 1 |  |  |

*AGI, acute gastrointestinal injury; GIDS, Gastrointestinal Dysfunction Score;* *ΔICI, Delta Integrated Calibration Index*


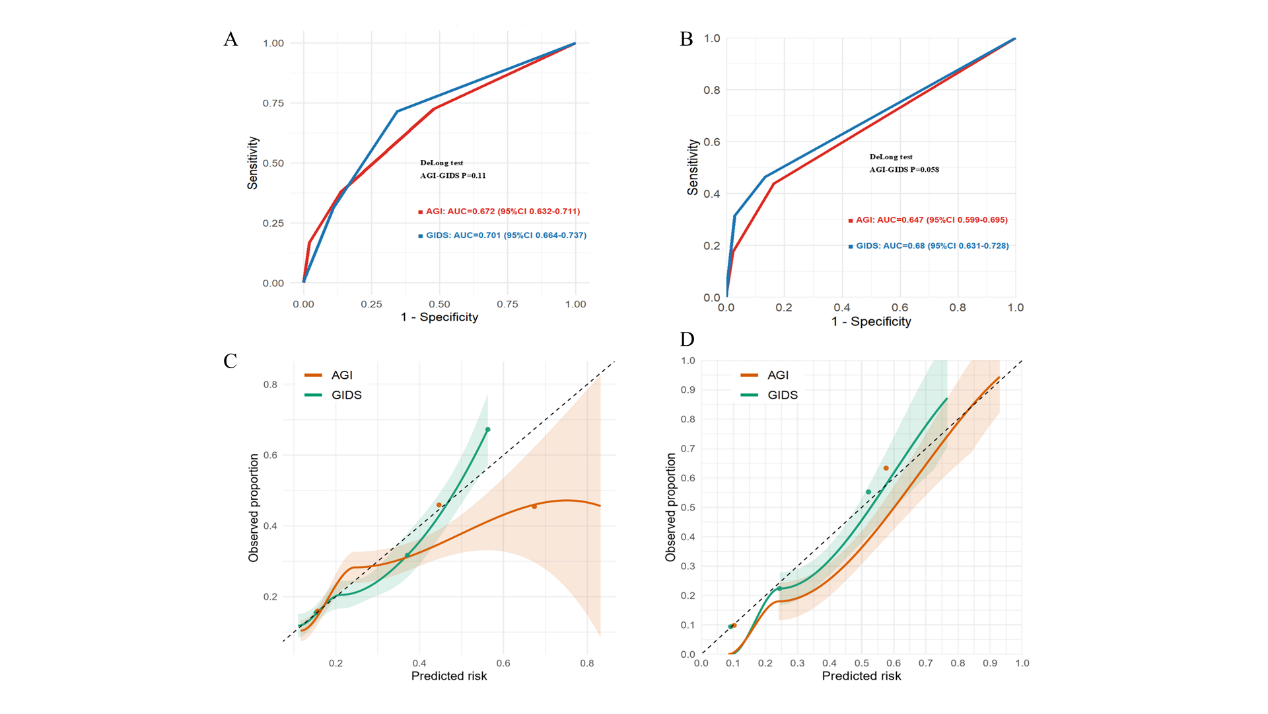


**Supplementary Figure S1. ROC curves and calibration plots of AGI grade and GIDS for 28-day mortality in two datasets.**
*(A, B) ROC curves; (C, D) calibration plots.
(A, C) FJLU cohort dataset; (B, D) NEED trial dataset.*

**Supplementary Table S9 Statistical Analysis of Calibration Curves for the two cohorts after Adjustment for Confounding factors**

|  | FJLU Cohort t | | | | | | NEED Trial | | | | | |
| --- | --- | --- | --- | --- | --- | --- | --- | --- | --- | --- | --- | --- |
|  | intercept | p | Slope | p | ΔICI  (GIDS-AGI) | p | intercept | p | Slope | p | ΔICI  (GIDS-AGI) | p |
| AGI grade | 0 | 1 | 1 | 1 | 0.009  （-0.026-0.010） | 0.30 | 0 | 1 | 1 | 1 | 0.002  （-0.010, 0.016） | 0.77 |
| GIDS | 0 | 1 | 1 | 1 |  |  | 0 | 1 | 1 | 1 |  |  |

*AGI, acute gastrointestinal injury; GIDS, Gastrointestinal Dysfunction Score;* ΔICI, *Delta Integrated Calibration Index*


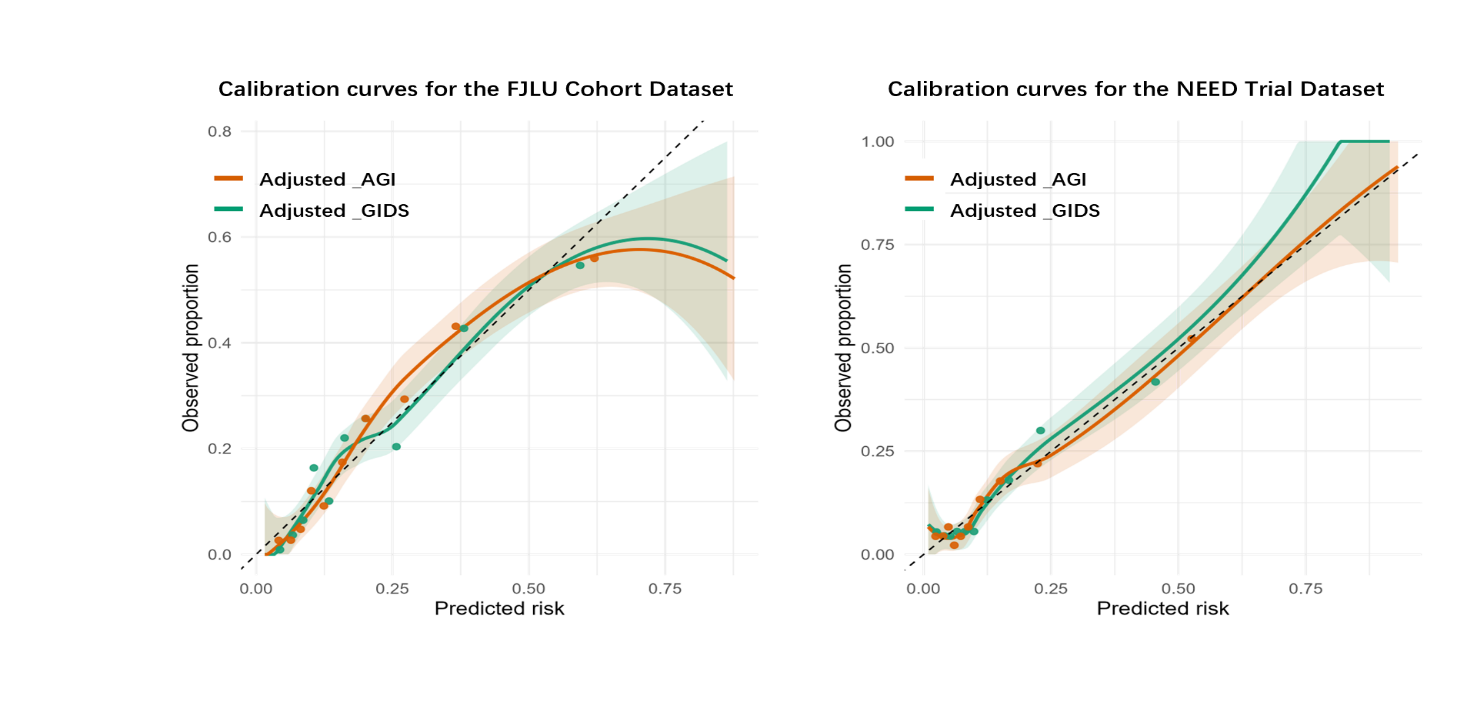


**Supplementary Figure S2. Confounder-adjusted calibration curves of AGI grade and GIDS for predicting 28-day mortality in two datasets.** *Adjusted_AGI: multivariable model including AGI grade adjusted for potential confounders.
Adjusted_GIDS: multivariable model including GIDS adjusted for potential confounders.
AGI, acute gastrointestinal injury; GIDS, Gastrointestinal Dysfunction Score.*

2.3.2 **Subgroup analyses of the discriminative performance of AGI grade and GIDS**

Subgroup analyses stratified by age, BMI, APACHE II, SOFA, and Mnutric. Forest plots summarizing AUCs across all prespecified subgroups are provided in *Supplementary Figures S3 and S4,* and subgroup-specific ROC curves are shown in *Supplementary Figures S5 and S6*.


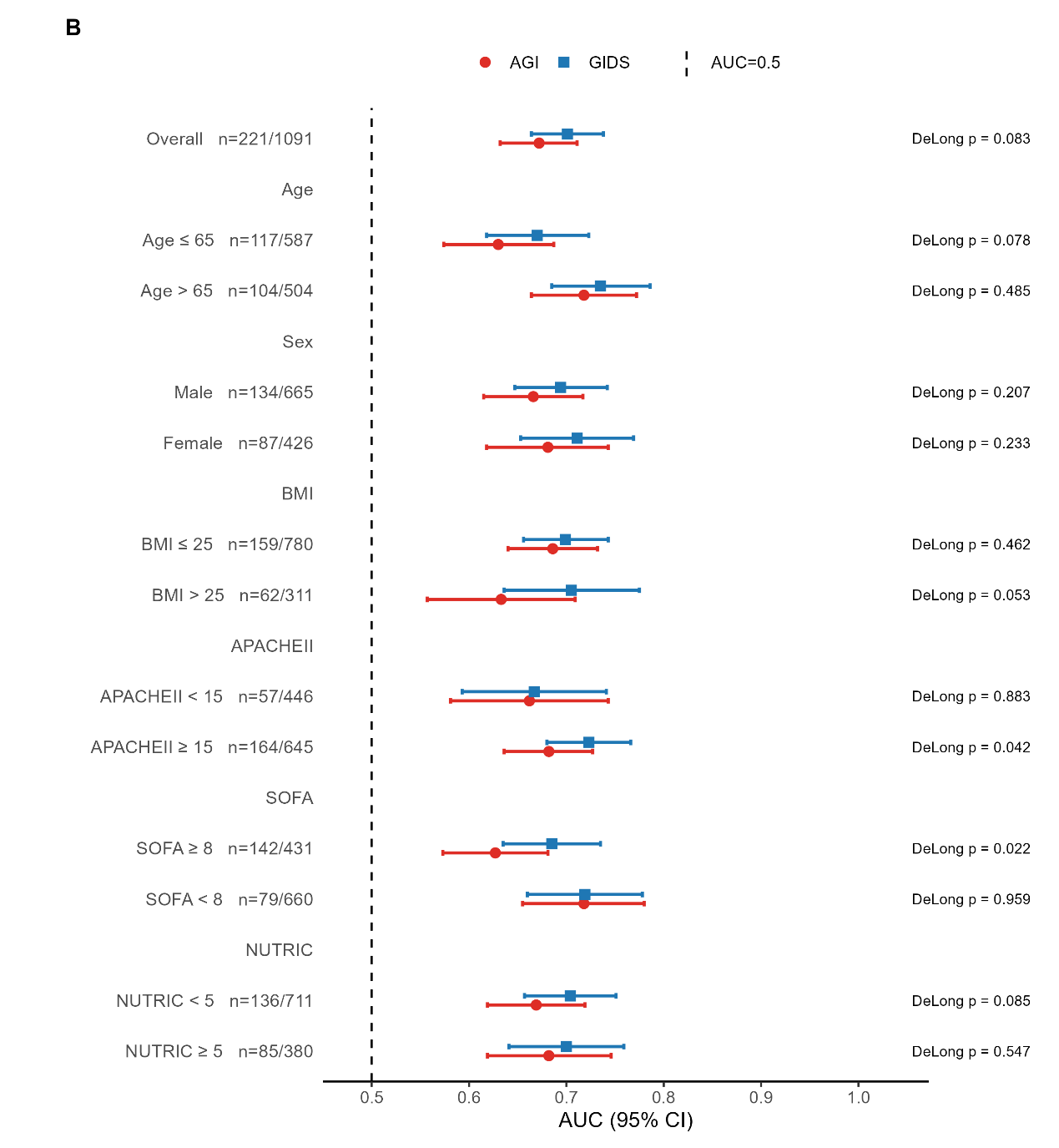
**Supplementary** **Figure S3 Forest plot of AUC for 28-day mortality of AGI and GIDS in each subgroup of the FJLU Cohort Dataset**

*BMI, body mass index; AGI, acute gastrointestinal injury; GIDS, Gastrointestinal Dysfunction Score; APACHE II, Acute physiology and chronic health evaluation II; SOFA, Sequential organ failure assessment; NUTRIC, modified Nutrition Risk in the Critically Ill; AUC, area under the receiver operating characteristic curve; CI, confidence interval*


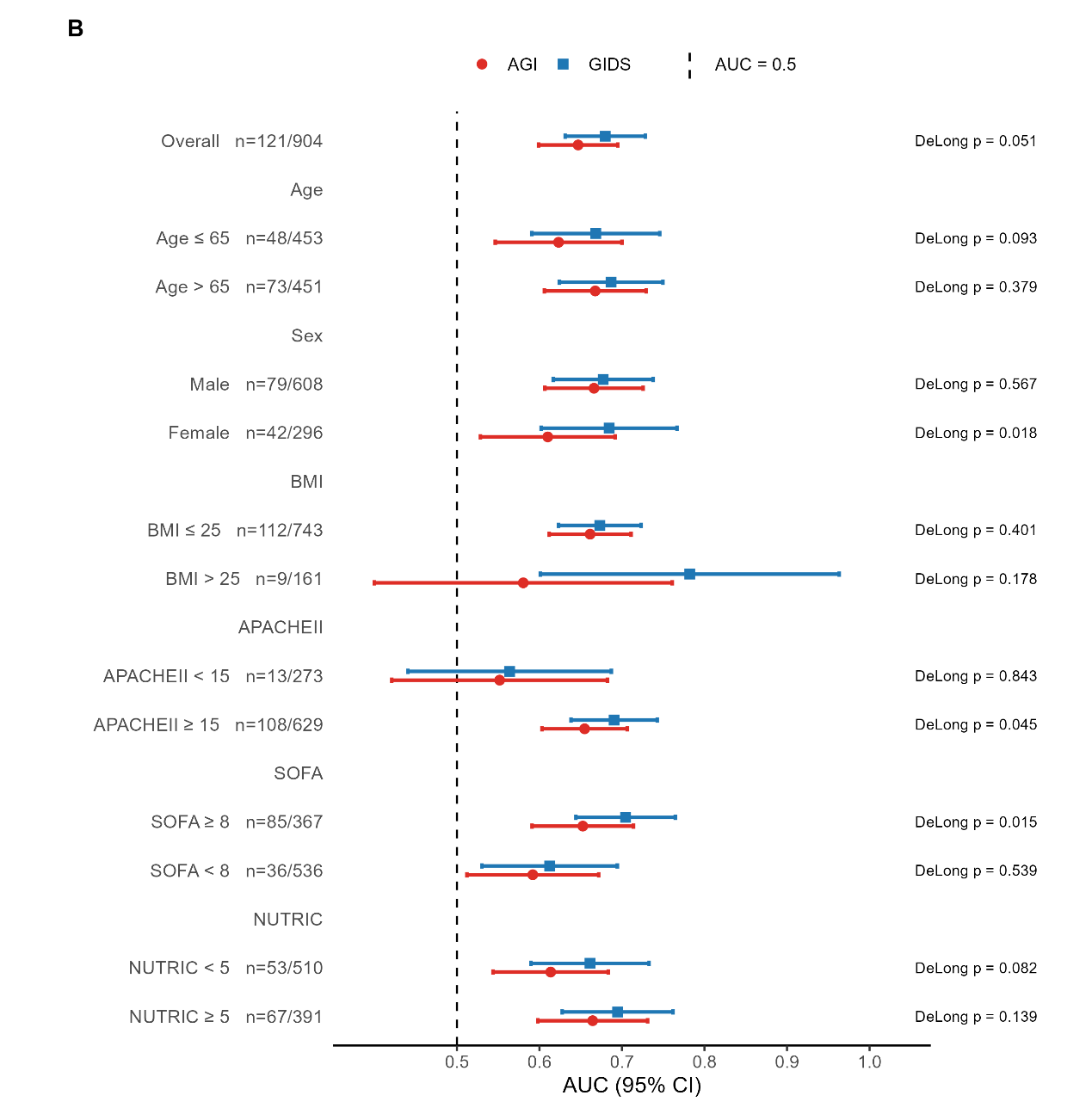


**Supplementary Figure S4 Forest plot of AUC for 28-day mortality of AGI and GIDS in each subgroup of the NEED Trial Dataset**

*BMI, body mass index; AGI, acute gastrointestinal injury; GIDS, Gastrointestinal Dysfunction Score; APACHE II, Acute physiology and chronic health evaluation II; SOFA, Sequential organ failure assessment; NUTRIC, modified Nutrition Risk in the Critically Ill; AUC, area under the receiver operating characteristic curve; CI, confidence interval*


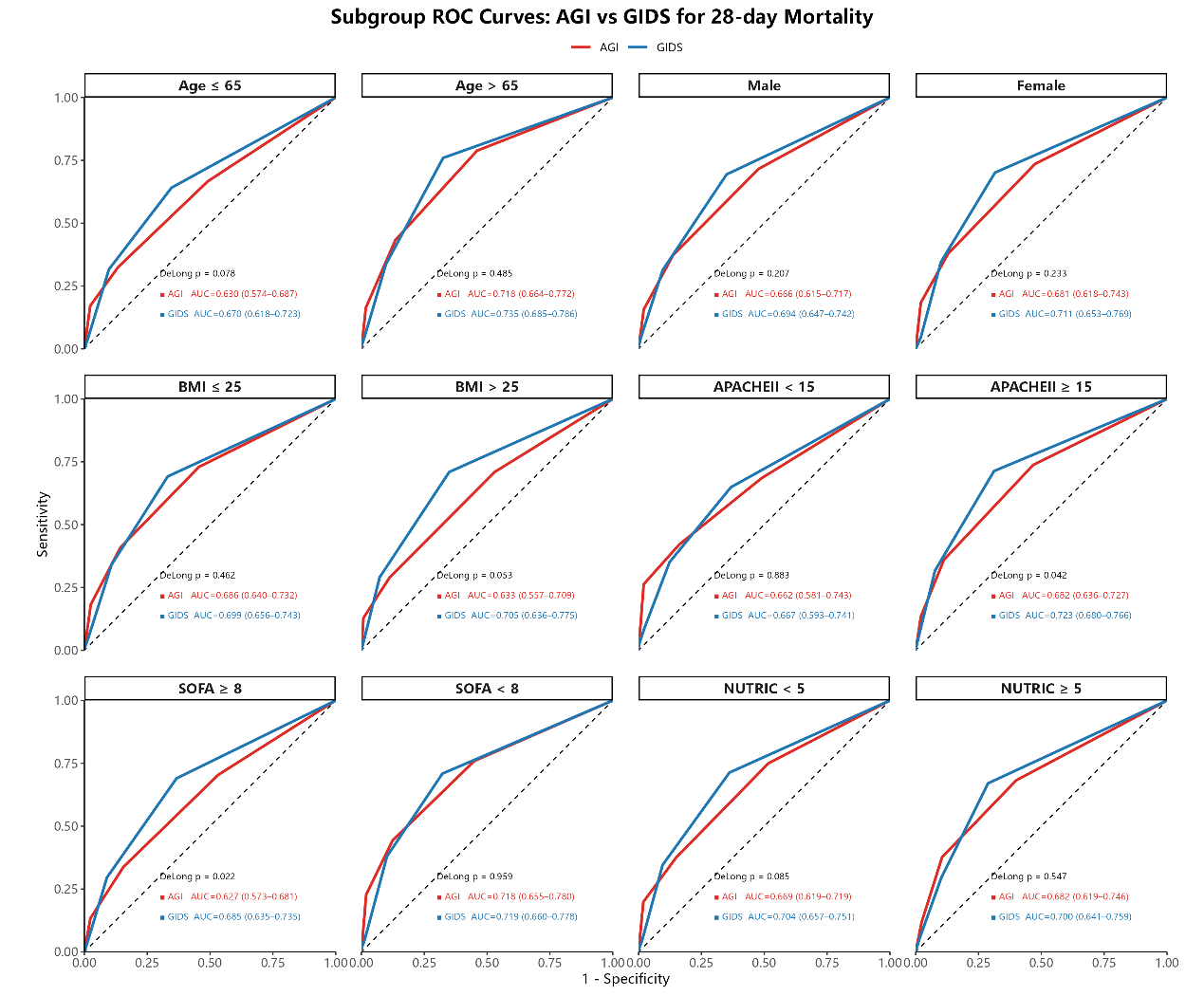
 **Supplementary Figure S5. Subgroup ROC curves comparing AGI grade and GIDS for predicting 28-day mortality in the FJLU cohort.**

*BMI, body mass index; AGI, acute gastrointestinal injury; GIDS, Gastrointestinal Dysfunction Score; APACHE II, Acute physiology and chronic health evaluation II; SOFA, Sequential organ failure assessment; mNUTRIC, modified Nutrition Risk in the Critically Ill; AUC, area under the receiver operating characteristic curve; CI, confidence interval*


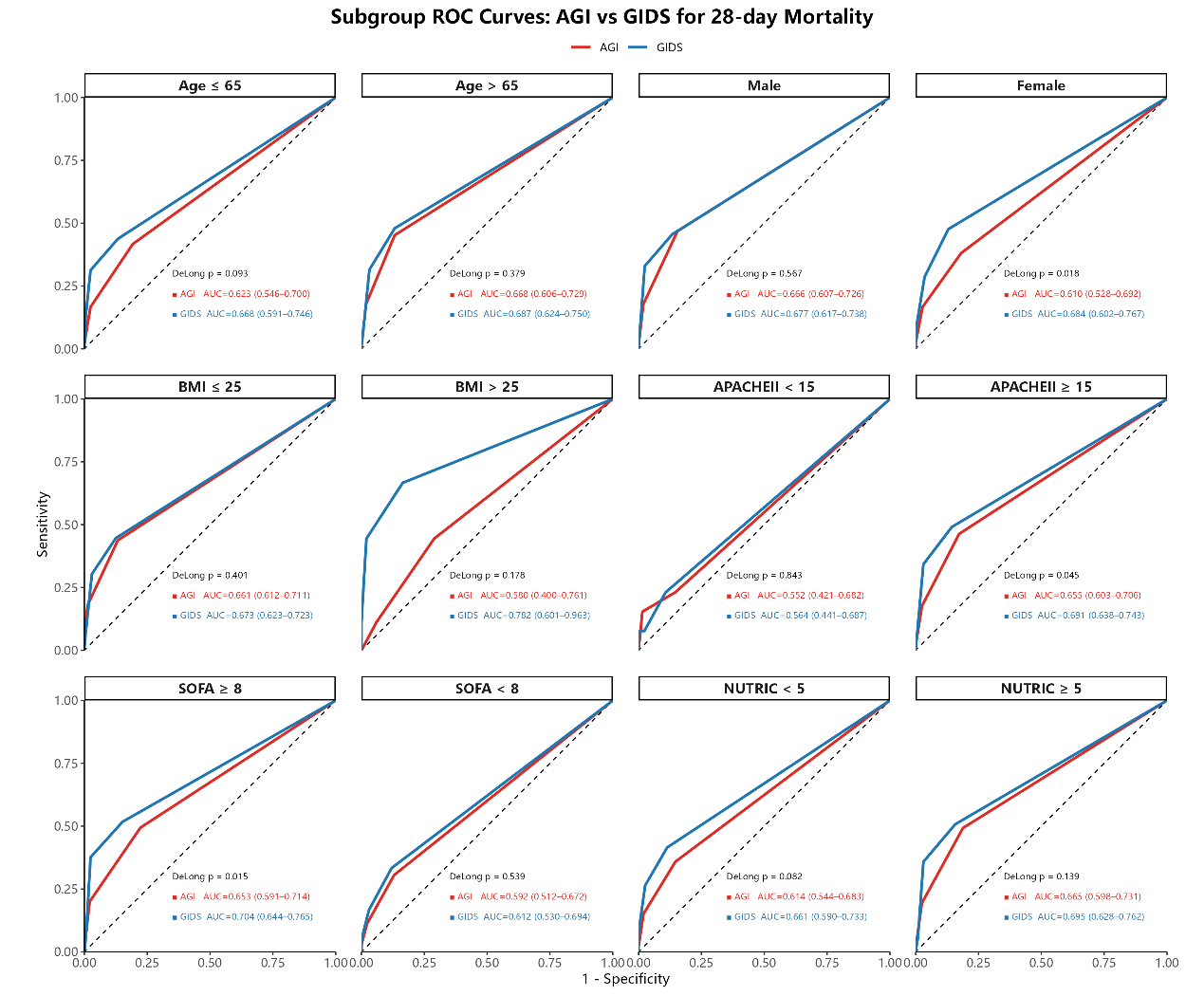
 **Supplementary Figure S6. Subgroup ROC curves comparing AGI grade and GIDS for predicting 28-day mortality in the NEED cohort.**

*BMI, body mass index; AGI, acute gastrointestinal injury; GIDS, Gastrointestinal Dysfunction Score; APACHE II, Acute physiology and chronic health evaluation II; SOFA, Sequential organ failure assessment; mNUTRIC, modified Nutrition Risk in the Critically Ill; AUC, area under the receiver operating characteristic curve; CI, confidence interval*


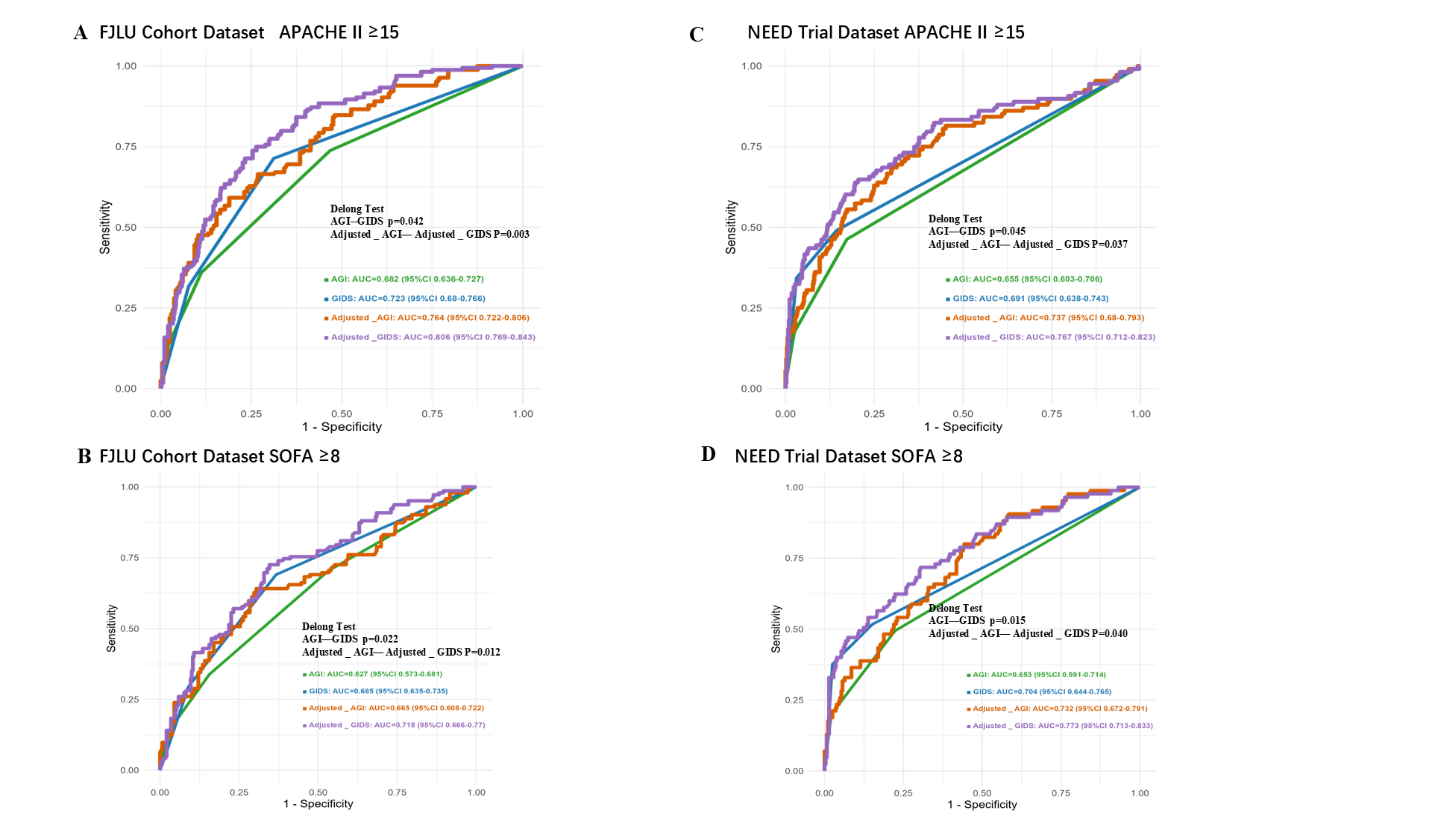


**Supplementary Figure S7. Confounder-adjusted ROC curves comparing AGI grade and GIDS for predicting 28-day mortality in patients with APACHE II ≥15 and SOFA ≥8 in two cohorts.**

*APACHE II, Acute physiology and chronic health evaluation II; SOFA, Sequential organ failure assessment; AGI, acute gastrointestinal injury; GIDS, gastrointestinal dysfunction score; ROC,receiver operating characteristic curve; AUC, Area Under the Curve; CI, confidence interval; Adjusted_AGI: multivariable model including AGI grade, adjusted for potential confounders. Adjusted_GIDS: multivariable model including GIDS, adjusted for potential confounders.*

*
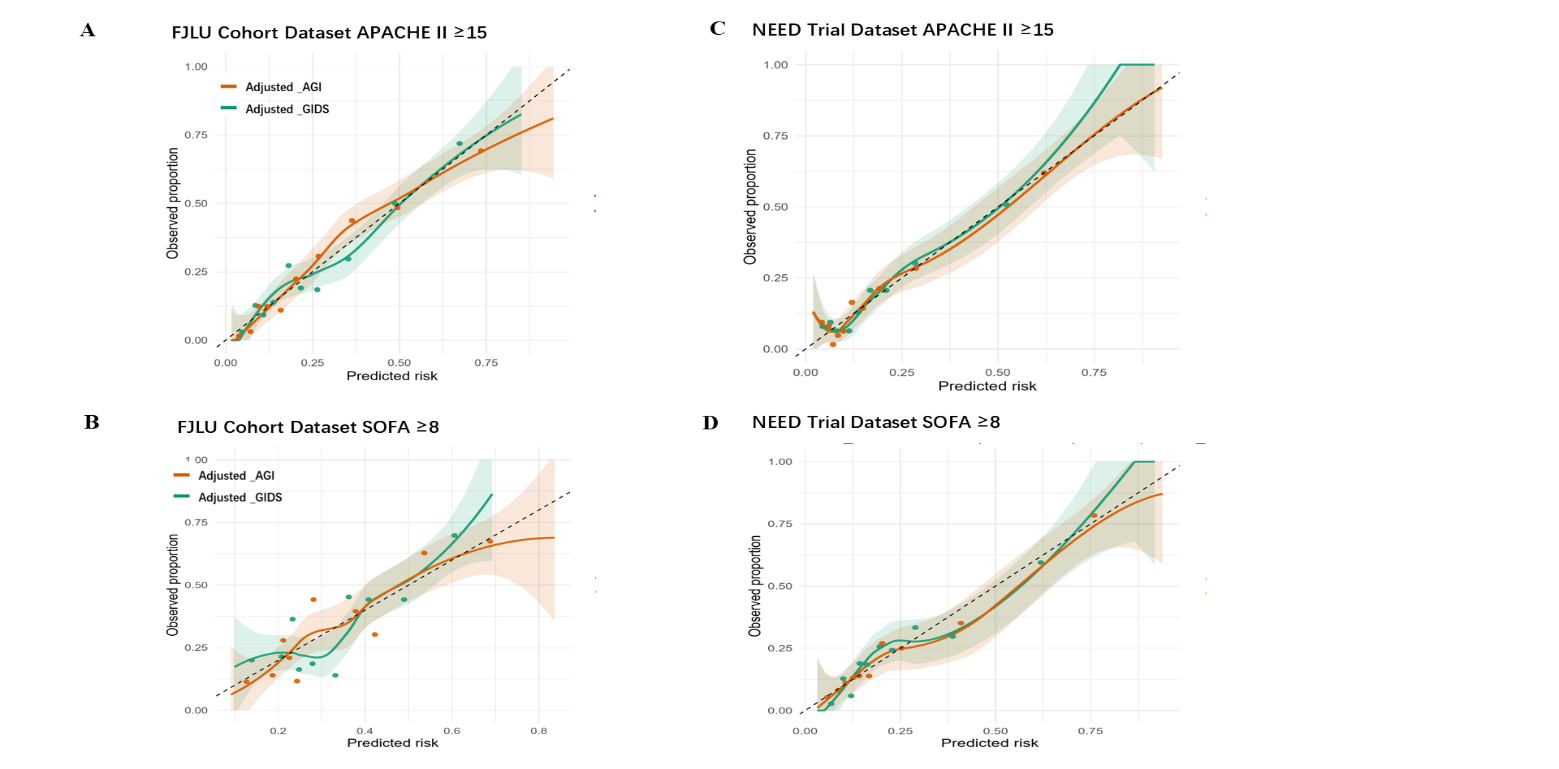
*

**Supplementary Figure S8 Calibration Curves After Confounder Adjustment in APACHE II ≥15 and SOFA ≥8 Subgroups in the two cohorts.**

*APACHE II, Acute physiology and chronic health evaluation II; SOFA, Sequential organ failure assessment; Adjusted_AGI: multivariable model including AGI grade, adjusted for potential confounders. Adjusted_GIDS: multivariable model including GIDS, adjusted for potential confounders.*

**Supplementary Table S10. Confounder-adjusted calibration of AGI grade and GIDS for 28-day mortality in patients with APACHE II ≥15 in two cohorts.**

| **FJLU Cohort Dataset (APACHE II ≥15)** | | | | | | | **NEED Trial Dataset (APACHE II ≥15)** | | | | | |
| --- | --- | --- | --- | --- | --- | --- | --- | --- | --- | --- | --- | --- |
|  | **intercept** | **p** | **Slope** | **p** | **ΔICI(GIDS-AGI)** | **p** | **intercept** | **p** | **Slope** | **p** | **ΔICI(GIDS-AGI)** | **p** |
| **AGI grade** | **0** | **1** | **1** | **1** | **0.001（-0.028, 0.025）** | **0.958** | **0** | **1** | **1** | **1** | **0.001（-0.014, 0.017）** | **0.824** |
| **GIDS score** | **0** | **1** | **1** | **1** |  |  | **0** | **1** | **1** | **1** |  |  |
| *AGI, acute gastrointestinal injury; GIDS, Gastrointestinal Dysfunction Score;* ΔICI, *Delta Integrated Calibration Index, APACHE II, Acute physiology and chronic health evaluation II; SOFA, Sequential organ failure assessment;*  **Supplementary Table S11. Confounder-adjusted calibration of AGI grade and GIDS for 28-day mortality in patients with SOFA ≥8 in two cohorts.** | | | | | | | | | | | | |
| **FJLU Cohort Dataset (SOFA ≥8)** | | | | | | | **NEED Trial Dataset (SOFA ≥8)** | | | | | |
|  | **intercept** | **p** | **Slope** | **p** | **ΔICI(GIDS-AGI)** | **p** | **intercept** | **p** | **Slope** | **p** | **ΔICI(GIDS-AGI)** | **p** |
| **AGI grade** | **0** | **1** | **1** | **1** | **0.0157（-0.021-0.052）** | **0.388** | **0** | **1** | **1** | **1** | **0.011（-0.014, 0.039）** | **0.418** |
| **GIDS score** | **0** | **1** | **1** | **1** |  |  | **0** | **1** | **1** | **1** |  |  |

*AGI, acute gastrointestinal injury; GIDS, Gastrointestinal Dysfunction Score; ΔICI, Delta Integrated Calibration Index, APACHE II, Acute physiology and chronic health evaluation II; SOFA, Sequential organ failure assessment;*

**3.3 Comparison of the Associations of AGI Grade and GIDS with ICU-free days at day 28**

To further examine the association of AGI grade and GIDS with ICU-free days to day 28, we performed multivariable linear regression analyses as a supplementary adjusted analysis. Model assumptions were assessed using residual plots, Q–Q plots, the Breusch–Pagan test, and the Shapiro–Wilk test (Supplementary Figure S9). Because heteroscedasticity was detected in the adjusted models, regression analyses were conducted using HC3 robust standard errors. Models were adjusted for age, SOFA score, mechanical ventilation, highest lactate within the first 24 hours, and BMI. AGI grade and GIDS were first evaluated in separate covariate-adjusted models and then entered simultaneously into joint models. Wald tests were used to compare the regression coefficients of AGI grade and GIDS in the joint models. Detailed regression results are shown in Supplementary Table S12.


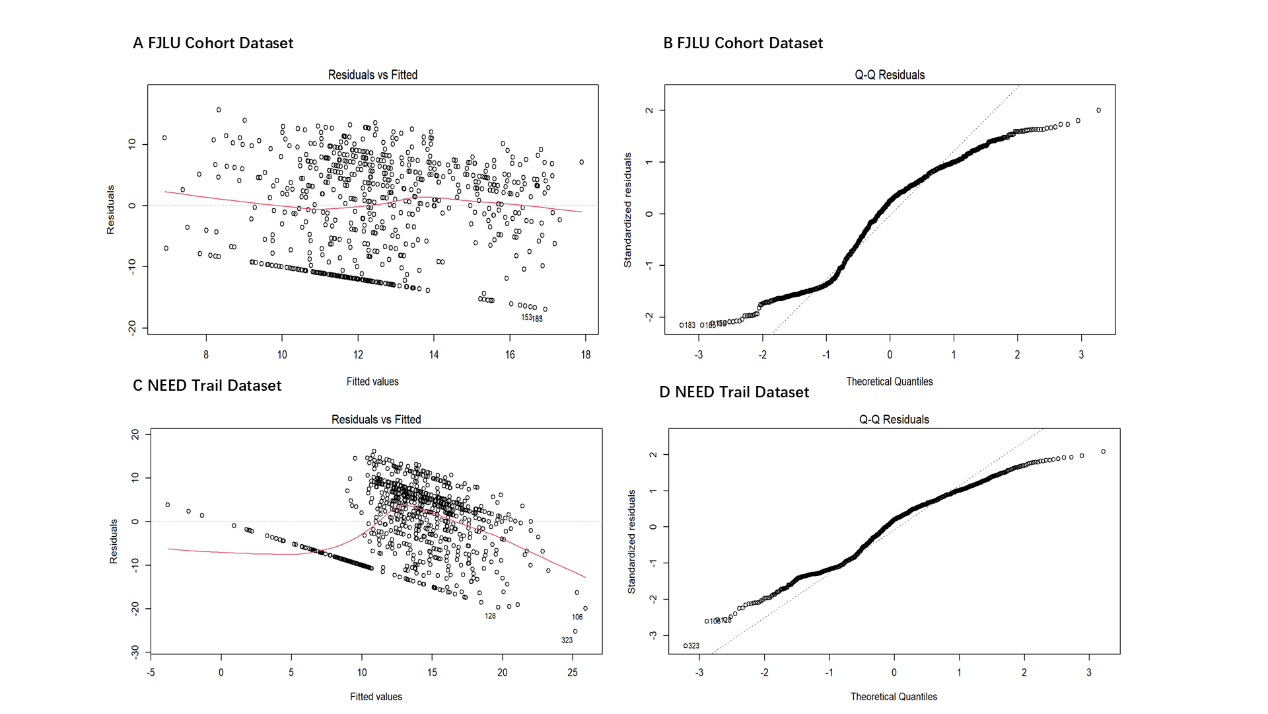


**Supplementary Figure S9 Residual diagnostics for the linear model of AGI, GIDS, covariates, and ICU-free days at day 28**

*FJLU :studentized Breusch-Pagan test P=0.002, Shapiro–Wilk test for residuals p＜0.001*

*NEED: studentized Breusch-Pagan test P=0.009, Shapiro–Wilk test for residuals p＜0.001*

**Supplementary Table S12. Multivariable linear regression of AGI and GIDS in relation to ICU-free days to day 28 with robust standard errors.**

| Model | FJLU Cohort | | NEED Trial | |
| --- | --- | --- | --- | --- |
|  | B coefficient (95% CI) | P value | B coefficient (95% CI) | P value |
| AGI+Covariates | -0.80 (-1.47– -0.13) | 0.019 | -0.923(-2.08– -0.23) | 0.116 |
| GIDS+ Covariates | -0.77 (-1.47– -0.07) | 0.030 | -1.13(-2.06 – -0.20) | 0.002 |
| AGI+GIDS+Covatiates (Joint model) | AGI: -0.60 (-1.35–-0.15) | 0.120 | AGI:-0.29(-1.59– -1.01) | 0.663 |
|  | GIDS: -0.50 (-1.28–-0.28) | 0.207 | GIDS:-1.00(-2.06 – -0.06) | 0.065 |
| Difference GIDS- AGI (Joint model) | 0.10 (-1.21–1.40) | 0.887 | -0.71 (-2.76–1.33) | 0.495 |

GIDS, Gastrointestinal Dysfunction Score; AGI, acute gastrointestinal injury; CI, confidence interval

**References**

1. Reintam Blaser A, Malbrain ML, Starkopf J, Fruhwald S, Jakob SM, De Waele J, et al. Gastrointestinal function in intensive care patients: terminology, definitions and management. Recommendations of the ESICM Working Group on Abdominal Problems. Intensive Care Med. 2012;38(3):384-94.
